# Puumala orthohantavirus: prevalence, biology, disease, animal models and recent advances in therapeutics development and structural biology

**DOI:** 10.3389/fimmu.2025.1575112

**Published:** 2025-05-08

**Authors:** Alina Tscherne, Pablo Guardado-Calvo, Jordan J. Clark, Robert Krause, Florian Krammer

**Affiliations:** ^1^ Ignaz Semmelweis Institute, Interuniversity Institute for Infection Research, Medical University of Vienna, Vienna, Austria; ^2^ G5 Unit Structural Biology of Infectious Diseases, Institut Pasteur, Université Paris Cité, Paris, France; ^3^ Department of Microbiology, Icahn School of Medicine at Mount Sinai, New York, NY, United States; ^4^ Center for Vaccine Research and Pandemic Preparedness (C-VaRPP), Icahn School of Medicine at Mount Sinai, New York, NY, United States; ^5^ Department of Internal Medicine, Division of Infectious Diseases, Medical University of Graz, Graz, Austria; ^6^ Department of Pathology, Molecular and Cell-Based Medicine, Icahn School of Medicine at Mount Sinai, New York, NY, United States

**Keywords:** Puumala orthohantavirus, animal models, vaccine research, antiviral treatment, glycoprotein, nephropathia epidemica

## Abstract

Puumala orthohantavirus (PUUV) is an emerging zoonotic virus that was first discovered in the Puumala region of Finland in the early 1980s and is the primary etiological agent of nephropathia epidemica (NE), a milder form of a life-threatening disease known as hemorrhagic fever with renal syndrome (HFRS). PUUV and other members of the Old World hantaviruses (OWHVs) predominantly circulate in rodents or insectivores across Eurasia, accounting for several thousand of reported HFRS cases every year (with many more unreported/misdiagnosed cases suspected). The rodent reservoir of PUUV is the common bank vole (*Myodes (M.) glareolus*), and transmission of the virus to humans occurs via inhalation of contagious aerosols and through contact with contaminated droppings or urine. Although PUUV is the subject of extensive research, due to its potential to cause severe disease outcomes in humans and its considerable economic and social impact, neither licensed vaccines nor specific antiviral treatments are available against PUUV. However, many important advancements have been made in terms of PUUV research over the last years. This included the elucidation of its glycoproteins, the discovery of broadly neutralizing hantavirus antibodies as therapeutic candidates and expanded research on the mRNA vaccine technology which will likely enable the development of strong PUUV vaccine candidates in the near future. Currently, there is still a lack of suitable animal models for the preclinical evaluation of experimental vaccines and antivirals, which hampers vaccine and antiviral development. Current attempts to decrease hantavirus-associated human infections rely primarily on prevention and countermeasures for rodent control, including reduced contact to droppings, saliva and urine, and disinfection of areas that are contaminated with rodent excreta. Here, we review these recent advances and other aspects including PUUV prevalence, virus biology, diagnosis and clinical features, and current animal models for vaccine and treatment development.

## 1 Introduction

Hantaviruses are a diverse family of single-stranded, trisegmented RNA viruses within the order Bunyavirales. Currently, Bunyavirales encompass eight genera *(Agnathovirus, Loan virus, Actinovirus, Percilovirus, Mobatvirus, Thottimvirus, Reptillovirus and Orthohantavirus)* (1, 2), which are known to infect different rodents and insectivores, with each strain being specific for a certain host species (3). The genus orthohantavirus includes species which vary in their geographic distribution and are capable of causing asymptomatic or mild to severe/lethal disease outcomes in humans. Generally, hantaviruses are classified into New World hantaviruses (NWHVs) and Old World hantaviruses (OWHVs) according to the geographic location of their respective rodent reservoir and the type of clinical manifestation upon infection of humans (4). NWHVs, such as Sin Nombre orthohantavirus (SNV) or Andes orthohantavirus (ANDV), mainly affect the human lung, causing a disease called hantavirus cardiopulmonary syndrome (HCPS) (5), and circulate in North and South America. OWHVs, such as Dobrava-Belgrade orthohantavirus (DOBV), Hantaan orthohantavirus (HTNV) and Puumala orthohantavirus (PUUV), mainly affect human kidneys, causing a disease called hemorrhagic fever with renal syndrome (HFRS) (6), and predominantly circulate in Eurasia. Furthermore, PUUV is associated with a third clinical phenotype, termed as nephropathia epidemica (NE) (7, 8), which is a less severe and milder form of HFRS. The case fatality rate (CFR) of PUUV infections ranges between 0.1-0.4% (9, 10), which is relatively low compared to the estimated high CFR of infections with NWHVs (30-60%) (11). Although most patients fully recover from an acute PUUV infection after weeks or months (12), several long-term sequelae (e.g., glomerular hyperfiltration, hypertension, stroke) (13) are observed. In addition, studies have indicated an increased risk to develop lymphatic/hematopoietic malignancies within the first years after recovering from a PUUV infection (14, 15).

Unlike other members of the order *Bunyavirales*, hantaviruses are not transmitted via obligate intermediate vectors, such as ticks, mosquitoes, flies or arthropods. However, they are directly or indirectly transmitted via hosts during close interactions, via inhalation of infectious aerosols or via contact with droppings or urine of infected animals (16–18). It has been shown that hantaviruses are more infectious via parental injection than aerosol transmission, thus, bite wounds and scratches caused by rodents present a risk for transmission that needs to be taken into account (19). Furthermore, observations of experimental ANDV and PUUV infections in Syrian hamsters indicate a potential transmission via the intragastric route (20, 21), thus, consumption of hantavirus contaminated food might be another conceivable way of transmission. The risk of direct human-to-human transmission of hantaviruses at this point is relatively low and almost neglectable, as humans are mostly dead-end hosts for the virus (2). So far, virus transmission from infected to naïve individuals has only been reported during ANDV-caused HCPS cases in Argentina (22, 23). In addition, one suspicious case of PUUV transmission via blood products in Finland has been reported recently (24) and mother-to-child transmission of ANDV through breast milk in Chile (25).

However, several questions regarding transmission to and pathogenesis in humans, but also the mechanisms of replication in both, humans and rodent hosts, including entry and tissue and organ tropism, still remain to be answered. Given the broad nature of this topic, it is beyond the scope of this review to describe all aspects of PUUV in-depth. Rather we aim to provide fundamental information about PUUV prevalence, its natural rodent reservoir, its viral biology, its diagnosis and clinical outcome, and treatment development, with an emphasis on current animal models and vaccine research.

## 2 PUUV prevalence and epidemiology

The initial discovery of hantaviruses dates back to in the 1950s during the Korean war (1951–1953) (7), where more than 3,000 military staff members suffered from a severe hemorrhagic fever disease of unknown origin. The etiologic agent of this hemorrhagic fever disease was isolated more than 25 years later, in 1978, from a striped field mouse (*Apodemus agrarius*) near the Hantan River in South Korea and was named HTNV (26). The second outbreak of severe hantavirus infections occurred in 1993 around the Four Corners region (New Mexico, Arizona, Utah, Colorado) in the United States of America. Individuals suffered from a hemorrhagic fever disease with pulmonary involvement, initially named Four Corners disease, later renamed as HCPS (27). In the same year, SNV was identified as the causative agent of the HCPS outbreak in the Four Corners region (28). In subsequent years, many other hantavirus species were identified globally in rodents or insectivores, e.g., ANDV in Argentina (1995) (29), or DOBV in Slovenia (1992) (30).

PUUV was first described (31) in the early 1980s, when the virus was detected in bank voles (*Myodes glareolus*) in the Puumala region of Finland. It is the causative agent of the vast majority of hantavirus infections in Europe within the last years (>98% of reported cases). Besides, other hantavirus species, such as TULV, DOBV, HTNV and Saaremaa orthohantavirus (SAAV), which persistently infect other rodents (e.g., *Microtus voles*, *Apodemus* mice), account for human HFRS infections in Europe (32–34). Eight PUUV lineages (4, 35–37) (**Table 1**) have been detected widely across Europe (with the exception of Southern Mediterranean coastal areas, British Isles, and the very Northern regions (8, 40)). However, only three European countries, Finland, Germany and Sweden, accounted for more than 85% of the annually reported cases (41–43) (**Tables 2**, **3**) in Europe within the last years. From 2010-2020, between 1,647 (in 2020) and 4,597 (in 2013) cases of hantavirus infections were reported in Europe (mean: 3,100). Interestingly, the reported case numbers follow a cyclical pattern, with a significant increase every two to three years. Notably, Finland has by far the highest infection rate per 100,000 population, ranging between 18.1 (in 2018) and 38.3 (in 2014) every year. Germany and Sweden, reporting the second and third most annual cases, respectively, have an infection rate per 100,000 population ranging between 0.2 (in 2013) and 3.5 (in 2012) and between 0.5 (in 2013) and 4.5 (in 2010), respectively (**Table 3**). However, epidemiological data are incomplete as many European countries do not report cases of hantavirus infection. In addition, low numbers of reported cases in regions with high hantavirus seroprevalence in the rodent population clearly demonstrate an underdiagnosis of hantavirus infections in Europe (44, 45). The incidence of PUUV infections in Europe varies considerably across time, from year to year, seasonally, and across countries, but also within each country (2, 32, 46) (**Tables 2**, **3**), and is strongly associated with the presence of its respective rodent host. Outbreaks of HFRS during spring and summer seasons are associated with close human contact with infected rodents during crop planting or harvesting, but also with increased travels of urban dwellers and camping tourists during the summer holiday season (42). In Northern Europe, hantavirus cases are frequently associated with close contact with infected rodents in the countryside (e.g., forest worker, soldiers) (27, 47).

**Table 1:** PUUV genetic lineages and their geographic location.

Lineage	Distribution	Reference
Alpe-Adrian (ALAD) lineage	Austria, Croatia, Hungary, Slovenia	(4, 35–39)
Central European (CE) lineage	Slovakia, Netherlands, Germany, Belgium, France	
Danish (DAN) lineage	Island of Fyne	
Finnish (FIN) lineage	Russian Karelia, Finland, Siberia (Omsk region)	
Latvian (LAT) lineage	Lithuania, Poland, Latvia	
North-Scandinavian (N-SCA) lineage	From Sweden (North) to Finland (Northwest)	
Russian (RUS) lineage	Pre-Ural Russia, Baltic countries (Estonia and Latvia)	
South-Scandinavian (S-SCA) lineage	From Norway to Sweden (central and South)	

**Table 2:** Distribution of hantavirus infection cases by country and year, EU/EEA, 2010-2020.

*Country*	*2010*	*2011*	*2012*	*2013*	*2014*	*2015*	*2016*	*2017*	*2018*	*2019*	*2020*
*Austria*	31	36	219	35	74	22	30	90	24	276	30
*Belgium*	212	190	62	21	74	44	38	123	85	57	9
*Bulgaria*	3	3	3	15	9	1	10	8	7	6	1
*Croatia*	NDR	NDR	154	6	209	10	31	389	18	191	17
*Cyprus*	NDR	NDR	NDR	0	0	0	0	0	0	0	0
*Czechia*	8	9	9	12	3	7	10	17	4	15	5
*Denmark*	NDR	NDR	NDR	NDR	NDR	NDR	NDR	NDR	NDR	NDR	NDR
*Estonia*	5	12	19	19	26	14	11	26	15	26	17
***Finland***	**1,443**	**1,834**	**841**	**1,685**	**2,089**	**1,463**	**1,663**	**1,246**	**999**	**1,256**	**1,164**
*France*	NDR	101	164	15	105	142	58	236	55	131	26
***Germany***	**2,016**	**305**	**2,825**	**161**	**574**	**829**	**282**	**1,731**	**235**	**1,535**	**229**
*Greece*	1	3	1	2	2	1	1	2	3	1	1
*Hungary*	11	7	8	2	6	9	7	16	6	13	4
*Iceland*	NDR	NDR	NDR	NDR	NDR	NDR	NDR	NDR	0	0	0
*Ireland*	0	0	1	1	0	0	0	0	0	0	0
*Italy*	NDR	NDR	NDR	0	0	NDR	0	0	0	0	0
*Latvia*	4	4	12	8	6	0	8	4	3	5	3
*Liechtenstein*	NDR	NDR	NDR	NDR	NDR	NDR	NDR	NDR	NDR	NDR	NDR
*Lithuania*	0	0	0	0	0	0	0	0	0	0	0
*Luxembourg*	0	0	23	0	3	13	1	15	0	8	0
*Malta*	0	0	0	0	0	0	0	0	0	0	0
*Netherlands*	0	0	0	1	1	1	2	6	1	0	0
*Norway*	21	39	13	19	42	11	10	26	21	11	12
*Poland*	6	8	3	8	54	6	8	14	11	9	3
*Portugal*	NDR	NDR	NDR	NDR	NDR	0	0	0	0	0	0
*Romania*	4	4	3	4	14	6	0	12	1	4	1
*Slovakia*	1	3	6	14	14	6	6	53	88	94	50
*Slovenia*	17	17	182	6	25	8	12	76	12	252	14
*Spain*	0	0	0	0	0	0	0	1	0	0	0
***Sweden***	**416**	**351**	**48**	**119**	**418**	**285**	**92**	**158**	**243**	**155**	**61**
*UK*	1	0	1	4	5	4	NDR	NDR	NDR	3	NDR
*Total EU-EEA*	4,200	2,926	4,597	2,157	3,753	2,897	2,280	4,249	1,831	4,048	1,647

Data obtained from ECDC (41–43); NDR, no data reported.

Highlighted in bold are the three countries with the highest number of reported hantavirus cases per year.

**Table 3:** Distribution of hantavirus infection rates per 100,000 population by country and year, EU/EEA, 2010-2020.

*Country*	*2010*	*2011*	*2012*	*2013*	*2014*	*2015*	*2016*	*2017*	*2018*	*2019*	*2020*
*Austria*	0.4	0.4	2.6	0.4	0.9	0.3	0.3	1.0	0.3	3.1	0.3
*Belgium*	2.0	1.7	0.6	0.2	0.7	0.4	0.3	1.1	0.7	0.5	0.1
*Bulgaria*	0.0	0.0	0.0	0.2	0.1	0.0	0.1	0.1	0.1	0.1	0.0
*Croatia*	NDR	NDR	3.6	0.1	4.9	0.2	0.7	9.4	0.4	4.7	0.4
*Cyprus*	NDR	NDR	NDR	0.0	0.0	0.0	0.0	0.0	0.0	0.0	0.0
*Czechia*	0.1	0.1	0.1	0.1	0.0	0.1	0.1	0.2	0.0	0.1	0.0
*Denmark*	NDR	NDR	NDR	NDR	NDR	NDR	NDR	NDR	NDR	NDR	NDR
*Estonia*	0.4	0.9	1.4	1.4	2.0	1.1	0.8	2.0	1.1	2.0	1.3
***Finland***	**27.0**	**34.1**	**15.6**	**31.1**	**38.3**	**26.7**	**30.3**	**22.6**	**18.1**	**22.8**	**21.1**
*France*	NDR	0.2	0.3	0.0	0.2	0.2	0.1	0.4	0.1	0.2	0.0
***Germany***	**2.5**	**0.4**	**3.5**	**0.2**	**0.7**	**1.0**	**0.3**	**2.1**	**0.3**	**1.8**	**0.3**
*Greece*	0.0	0.0	0.0	0.0	0.0	0.0	0.0	0.0	0.0	0.0	0.0
*Hungary*	0.1	0.1	0.1	0.0	0.1	0.1	0.1	0.2	0.1	0.1	0.0
*Iceland*	NDR	NDR	NDR	NDR	NDR	NDR	NDR	NDR	0.0	0.0	0.0
*Ireland*	0.0	0.0	0.0	0.0	0.0	0.0	0.0	0.0	0.0	0.0	0.0
*Italy*	NDR	NDR	NDR	0.0	0.0	NDR	0.0	0.0	0.0	0.0	0.0
*Latvia*	0.2	0.2	0.6	0.4	0.3	0.0	0.4	0.2	0.2	0.3	0.2
*Liechtenstein*	NDR	NDR	NDR	NDR	NDR	NDR	NDR	NDR	NDR	NDR	NDR
*Lithuania*	0.0	0.0	0.0	0.0	0.0	0.0	0.0	0.0	0.0	0.0	0.0
*Luxembourg*	0.0	0.0	4.4	0.0	0.5	2.3	0.2	2.5	0.0	1.3	0.0
*Malta*	0.0	0.0	0.0	0.0	0.0	0.0	0.0	0.0	0.0	0.0	0.0
*Netherlands*	0.0	0.0	0.0	0.0	0.0	0.0	0.0	0.0	0.0	0.0	0.0
*Norway*	0.4	0.8	0.3	0.4	0.8	0.2	0.2	0.5	0.4	0.2	0.2
*Poland*	0.0	0.0	0.0	0.0	0.1	0.0	0.0	0.0	0.0	0.0	0.0
*Portugal*	NDR	NDR	NDR	NDR	NDR	0.0	0.0	0.0	0.0	0.0	0.0
*Romania*	0.0	0.0	0.0	0.0	0.1	0.0	0.0	0.1	0.0	0.0	0.0
*Slovakia*	0.0	0.1	0.1	0.3	0.3	0.4	0.1	1.0	1.6	1.7	0.9
*Slovenia*	0.8	0.8	8.9	0.3	1.2	0.4	0.6	3.7	0.6	12.1	0.7
*Spain*	0.0	0.0	0.0	0.0	0.0	0.0	0.0	0.0	0.0	0.0	0.0
***Sweden***	**4.5**	**3.7**	**0.5**	**1.2**	**4.3**	**2.9**	**0.9**	**1.6**	**2.4**	**1.5**	**0.6**
*UK*	0.0	0.0	0.0	0.0	0.0	0.0	NDR	NDR	NDR	0.0	NDR
*EU-EEA*	1.2	0.7	1.1	0.4	0.8	0.6	0.5	1.0	0.4	0.8	0.4

Data obtained from ECDC (41–43); NDR, no data reported.

Highlighted in bold are the three countries with the highest infection rate per 100,000 population.

## 3 Natural reservoir

Hantaviruses have been detected in different families of rodents (e.g., *Muridae* and *Cricetidae*) (26), bats (e.g., *Vespertilionidae*, *Rhinolophidae*, and *Nycteridae*) (48) and insectivores (e.g., *Talpidae*, *Soricidae*) (49). They seem to be very strictly associated with one or very few closely related reservoir species and follow the distribution of the respective reservoir (8, 50). The main, and in Central Europe exclusive, reservoir for PUUV are common bank voles (*M. glareolus*), which are small rodents that are found in temperate and boreal forests (taiga) (51), but also in urban gardens, parks and hedges (46). Interestingly, genetically closely related PUUV species from Asia (Japan, China) have been found in vole species other than *M. glareolus*, but did not show any pathogenicity in humans (51, 52) so far.

There is in fact a strong relationship between the bank vole population density, PUUV prevalence, and the number of PUUV infections in humans in a specific area (53, 54). The population dynamics of bank voles change intra-and inter-annually within Europe and depend on climate changes (55) and variations in the landscape attributes (56), but also on other extrinsic factors, such as social behavior, the presence of predators (e.g., weasels) (57) or the availability of food (18). In temperate Europe rodent population increases mainly due to mast years, which occur when a substantial number of nuts from beech (*Fagus sylvatica*) or oak trees (*Quercus petraea* and *Q. robur*) pile up on the ground (58), providing sufficient nutrition for the rodents. Due to this substantial higher supply of food, the survival rate of the voles increases with an earlier breeding throughout the winter, causing a fluctuation of the bank vole population that can be 10-fold higher in these years compared to normal years (58, 59). In addition, studies have shown a correlation between an increase of bank vole abundance and an increase of beech fructification (60) or bilberry production (61) the year prior. Infections of bank voles and other rodents with hantaviruses apparently causes a prolonged or persistent infection (8, 62), which can last several months (46, 63) and is characterized by a subclinical or asymptomatic course (63). Apparent symptoms have not been detected in PUUV infected rodents; however, host survival and maturation (64) might be impaired. Infected rodents shed the virus through feces, urine and saliva (65, 66), causing subsequent infections of hosts via bites and scratches or contact with contagious excreta (67). Vertical transmission of the virus is less unlikely, as maternal antibodies protect the offspring (27). Studies reported a transient viraemia in infected bank voles (66, 68, 69), and infectious virus (66), PUUV antigen (62, 66, 68) or viral RNA (68, 69) could be detected several weeks or months after infection in various organs.

## 4 Virology

### 4.1 Virus structure and genome organization

**Figure 1:**
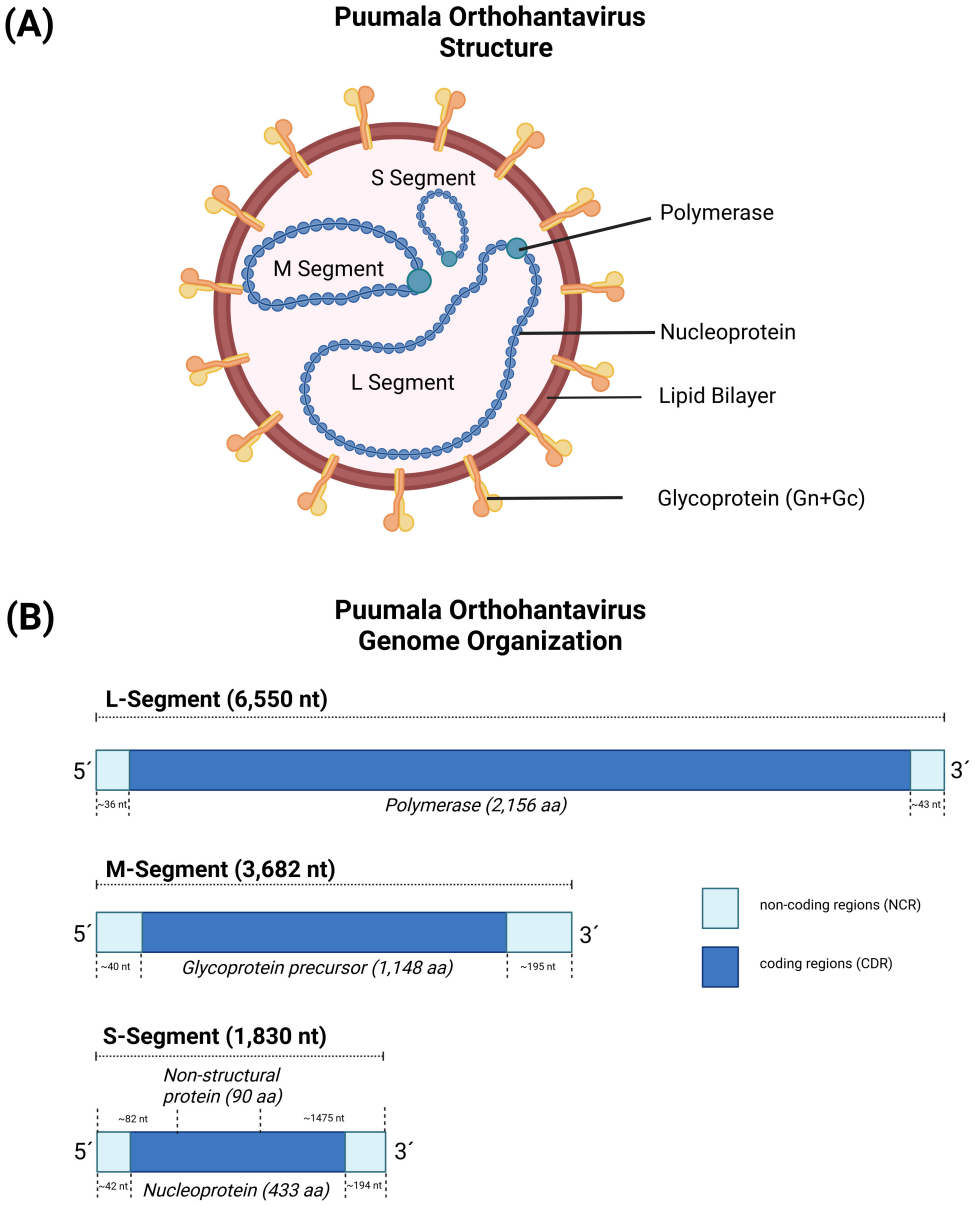
The virus particle and genome structure of Puumala orthohantavirus (PUUV). **(A)** The genome structure of orthohantaviruses, based on PUUV strain Sotkamo, accession numbers MN832782.1, MN832783.1, and MN832784.1 for the L, M, and S segment, respectively. **(B)** The S segment encodes for the nucleoprotein (433 aa) and the non-structural protein (90 aa), the M segment encodes for a glycoprotein precursor (1,148 aa), and L segment encodes for an RNA-dependent RNA polymerase (2,156 aa). Created with BioRender.com.

Generally, hantaviruses display a spherical to pleomorphic shape (70), with a diameter of the virions broadly ranging between 80-160 nm (71, 72). Virions are relatively stable and survive for a few days at room temperature and up to several weeks at 4°C and -20°C (8, 65). The negative-sense and tri-segmented RNA genome is found within a lipid bilayer-based, enveloped virion, comprising of a large segment (L), a medium segment (M) and a small segment (S) (**Figure 1A**) (73), which display different sizes among the hantaviruses. The PUUV L segment is ~ 6,550 nucleotides (nt) in size and encodes for a ~ 2,156 amino acid (aa) long L protein. The L protein is an RNA-dependent RNA polymerase (RdRp), which mediates transcription and replication of the viral RNA genome. The PUUV M segment is ~ 3,682 nt in size and encodes for a ~ 1,148 aa long precursor glycoprotein (GPC). GPC is co-translationally processed into two envelope proteins, Gn and Gc, which are important for binding to the respective host cell receptor and the subsequent entry of the virus (74). The PUUV S segment is ~ 1,830 nt in size and encodes for a ~ 433 aa long nucleoprotein (N), that encapsidates the viral RNA genome (75). PUUV, TULV and other hantavirus species infecting members of the family *Cricetidae* (e.g., lemmings, New World mice/rats) (73, 76) additionally encode for a non-structural (NSs) protein (PUUV NSs: ~90 aa in size), which is located on the S segment in an overlapping open reading frame (ORF) (**Figure 1B**) (73, 77), and expressed via leaky scanning. Leaky scanning is a wide spread process among viruses to express polycistronic RNA, in which scanning ribosomes skip the first start codon and initiate protein synthesis at downstream located start codons (78). NSs is thought to be a non-essential protein, however, if expressed, it plays a role in the viral pathogenesis and immune evasion of infected hosts (79).

The 5´and 3´ non-coding regions of the three segments have different lengths (**Figure 1B**), ranging between 40-50 nt (5´ of all three segments) to 300-700 nt (3´ of S and M segment). Interestingly, the very terminal part of the sequences (consensus sequence AUCAUCAUCUG) (80) is conserved within the hantaviruses and can form panhandle-like structures (81, 82), which is a hallmark of the respective genus and shared with other genera in the *Bunyavirales*. Sequence analysis of the L, M and S segments showed a high degree of genetic diversity between the different hantavirus species, which is most likely caused by the accumulation of point mutations in combination with deletions and insertions mainly in the non-coding areas of the viral RNA segments (81). In addition, there is evidence for genetic shift, which occurs through the reassortment and recombination of genome RNA segments (83, 84).

### 4.2 The structure of the glycoprotein shell

Hantaviruses have a glycoprotein shell that orchestrates all the steps required for viral entry and is the primary target for neutralizing antibodies (85–88). This shell is composed of the two membrane glycoproteins Gn and Gc, which are encoded on the M segment as a polyprotein precursor and processed to form a tetrameric (Gn/Gc)_4_ spike (89). Gn acts as a folding chaperone for Gc and regulates fusion timing, Gc is the protein responsible for mediating fusion. Gn is about 650 amino acids long and is composed of two globular regions (Gn^H^ and Gn^B^), two transmembrane (TM) regions and an intraviral domain. The second TM ends in a conserved motif that is cleaved to produce the Gc amino (N)-terminus (90). Gc is a class-II fusion protein of about 450 amino acids length, featuring an elongated ectodomain, a transmembrane region, and a short intraviral tail.

The structures of the Gn^H^/Gc, Gn^H^, Gn^B^ and Gc have been extensively studied by x-ray crystallography (87, 91–95) and the structure of the spike and its organization on the viral particle using cryo-electron microscopy (72, 96–98). The ectodomain of Gn is formed by three domains (A, B, C) and a membrane proximal region (MPR^N^). Domains A and B form the Gn^H^ region, which interact with Gc to stabilize its prefusion conformation and prevent premature association of Gc with cell membranes. Domain C and the MPR^N^ constitute the Gn^B^ region, which functions as the tetramerization domain of the spike. Gc is a class-II fusion protein formed by a central β-sandwich (domain I) flanked by domains II and III. Domain III connects to the transmembrane region via the stem, a flexible region that is about 30 amino acids long, and the Gc membrane proximal region (MPR^C^). Like other class-II fusion proteins, the prefusion complex Gn^H^/Gc dissociates at low pH, and Gc undergoes conformational changes that extend domain II toward the endosomal membrane to insert a hydrophobic region (termed the target membrane insertion surface (TMIS) in the hantavirus), reassemble into homotrimers, and refold domain III and the stem to approach the viral and endosomal membranes and induce fusion.

Despite these similarities, there are important structural and mechanistic differences between Gc and other class II fusion proteins. Notably, the TMIS of hantaviruses is composed of three flexible loops rather than a single rigid one. These loops coordinate to adopt two different conformations through an allosteric mechanism that is regulated by pH and the presence of Gn^H^ (92, 93). At neutral pH, Gn^H^/Gc forms a stable complex in which the side chains of three key hydrophobic residues (W766, Y745, and F900) are buried. In this conformation, the tip of domain II exposes a polar surface that cannot interact with membranes. At acidic pH, Gn^H^ dissociates from Gc, triggering a reorganization of the tip of domain II which exposes the key residues, allowing Gc to insert into the endosomal membrane. The precise mechanism controlling the reorganization of domain II remains unclear, but some evidence suggest that an unusual acidic hydrogen bond forms at low pH between the side chains of the conserved E757 and D759 residues and plays a critical role. The formation of this bond is required to structure the TMIS in the post-fusion conformation and the presence of Gn^H^ induces a major reorganization of the loop containing these residues, which prevents the formation of the acidic hydrogen bond.

**Figure 2:**
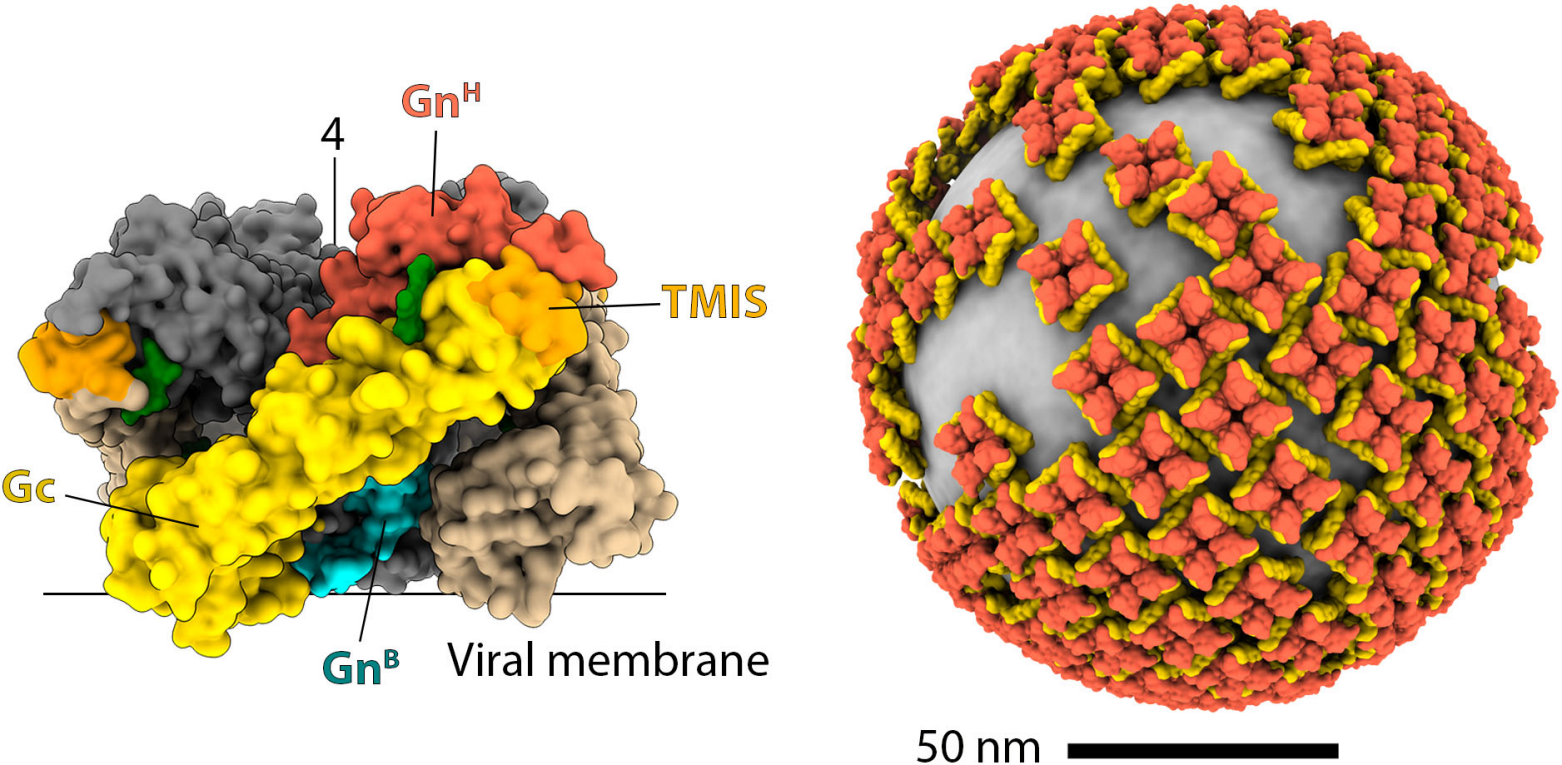
The organization of hantavirus spikes and the glycoprotein shell. The left panel shows a surface representation of the hantavirus spike in a side view. In the front protomer, Gn^H^, Gn^B^, and Gc are colored red, cyan, and yellow, respectively, as indicated. The TMIS is colored orange, and the N-glycans are shown in green. For clarity, the other protomers are colored differently: Gn in gray, and Gc in brown. The approximate positions of the viral membrane and the symmetry axis are indicated with lines. The right panel is a reconstruction of the hantavirus glycoprotein shell, with Gn and Gc colored red and yellow, respectively.

Another notable difference with other class-II fusion machineries is how Gn and Gc are organized on the viral surface. Cryo-electron microscopy (72, 96, 99) and biochemical studies (100, 101) revealed that the glycoprotein shell does not exhibit an icosahedral symmetry. Instead, it is composed of tetrameric (Gn/Gc)_4_ spikes, with four molecules of Gn at the center and four of Gc at the periphery, which interact laterally to form a grid-like pattern. This unique organization generates a topological problem because square-shaped spikes are incompatible with the formation of a closed, curved surface. Consequently, hantavirus particles display a distinctive pattern characterized by areas of ordered lattices coexisting with regions containing lattice-free spikes (**Figure 2**). Interestingly, a cryo-ET study (99) using the neutralizing antibody P4G2, which targets the interspike Gc/Gc interface and can only bind to isolated spikes, showed that this antibody induces the accumulation of isolated spikes on the viral surface. This finding reveals that the distribution between isolated and lattice-associated spikes is dynamic and can be influenced by the immune response. Along the same lines, ADI-42898, a cross-neutralizing antibody isolated from a patient infected with PUUV, binds to a quaternary epitope at the tip of Gc (85, 91). Structural modeling of ADI-42898 IgG molecules shows that they cannot bivalently bind to isolated spikes but cross-link neighboring tetramers within the virion lattice, likely promoting the accumulation of lattice-associated spikes. It remains to be seen if antibodies targeting the tip of domain II (like ADI-42898) interfere with the activity of those targeting the inter-spike regions (like P4G2).

Combining the cryo-ET map and x-ray crystallography models of Gn^H^/Gc and Gn^B^ tetramer produced a quasi-atomic model for the (Gn/Gc)_4_ spike (**Figure 2**) (92). In this model, Gn^B^ –the most conserved region of the polyprotein – is at the core of the spike, inaccessible for the immune system, and mediating most of the intra-spike interactions. Gn^H^, which is much more variable, is exposed at the membrane distal surface, where it makes extensive contacts with Gc. A distinctive feature that emerges from the model is that all N-linked glycans play a structural role, either stabilizing the interaction between Gn^H^/Gc or filling the internal cavities of the spike. Consistently, the removal of any of these glycans has been shown to impair the intracellular trafficking of the spike (102). Biochemical analysis revealed that the N-linked glycans remained of the high-mannose type in secreted particles (102). This observation suggests that spikes assemble early in the endoplasmic reticulum (ER), prior to transport to the Golgi apparatus, where glycan chains would otherwise undergo modification. Interestingly, some hantaviruses, such as DOBV, HTNV, and Thottapalayam virus (TPMV), possess additional N-glycosylation motifs that may help them to evade the immune system. However, the acquisition of new N-glycosylation sites appears to be a rare event in hantaviruses.

The structural studies conducted in recent years have provided important insights into how to design better immunogens and optimize the neutralizing activity of antibodies. Antibodies targeting Gn have potent neutralizing activity, but are serotype-specific, while antibodies targeting Gc are broadly neutralizing, but have weaker activity and tend to leave unneutralized fractions (85). The only cross-clade neutralizing antibody reported to date is ADI-42898, but it has reduced activity against ANDV. Structural studies (91) have shown that ADI-42898 recognizes a quaternary epitope that is only present in the prefusion conformation of the Gn/Gc heterodimer. Mechanistic studies have shown that ADI-42898 blocks viral membrane fusion by stapling together the Gn and Gc subunits, preventing them from dissociating at the acidic pH of the endosomes. These studies have also shown that ADI-42898 rapidly dissociates from the ANDV heterodimer at acidic pH, which limits its activity against this virus. *In vitro* affinity maturation experiments have identified mutations of this antibody that correct this defect and neutralize ANDV more effectively. Overall, these results suggest that stabilized heterodimers in the prefusion formation are better immunogens than Gn or Gc alone, leading to the development of various approaches to stabilize them, including the insertion of a linker between Gn and Gc, the design of disulfide bonds crosslinking Gn and Gc, or the introduction of mutations in Gc that interfere with the adoption of the post-fusion form (92).

### 4.3 Viral entry

*In vitro* studies indicated that integrins (β1-3) are potential candidate receptors, and complement factors (e.g., gC1qR/P32), decay acceleration factors (e.g., DAF/CD55) or protocadherin-1 are critical (co)-factors for viral attachment (103, 104) of NWHVs and OWHVs. Despite ongoing research in this field, the role of the suggested candidate receptors in pathogenesis and host range restriction is poorly understood [reviewed in (103)].

The primary targets for hantavirus replication in humans are macrophages, dendritic cells, (micro)vascular endothelial cells (69, 105), and pulmonary cells (106). Interestingly, *in vitro* studies have shown that the hantavirus tropism for cells belonging to the mononuclear phagocyte system is not exclusively limited to human, as also dendritic cells of the rodent reservoir can be productively infected (107). Apparently, the viral replication does not directly kill or damage the cells and the vascular endothelium; however the endothelial barrier integrity is impaired due to excessive and uncontrolled innate and adaptive immune responses (108–110).

### 4.4 Replication cycle

**Figure 3:**
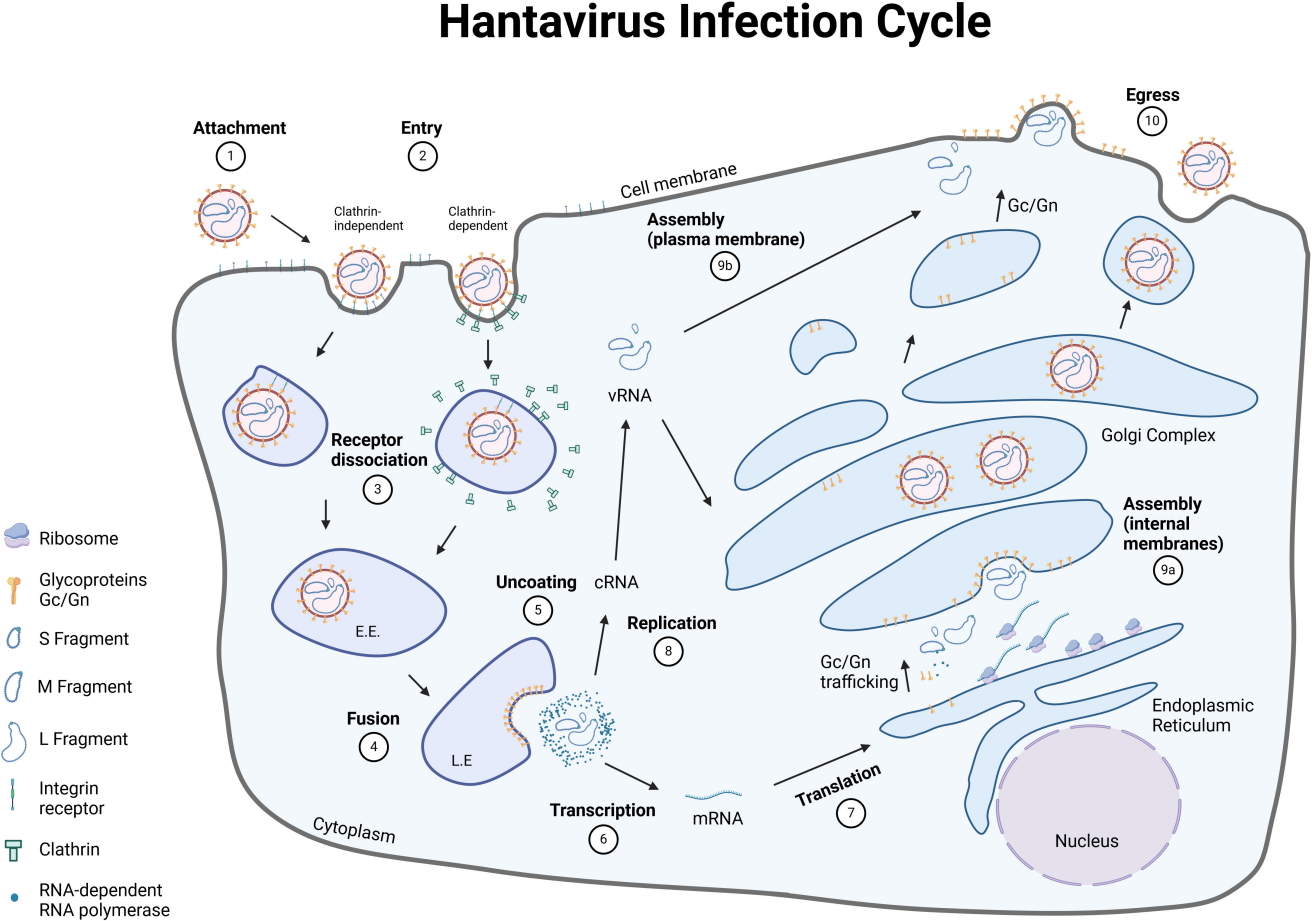
Hantavirus life cycle. The hantavirus life cycle consists of ten major steps, that are necessary to release new viral particles. [1] Hantaviruses bind to their respective receptor on the surface of the host cell with the envelope glycoproteins Gn/Gc. [2] Entry of the viral particles occur either via clathrin-dependent (OWHVs, e.g., PUUV) or clathrin-independent endocytosis (NWHVs). [3] The viral glycoproteins dissociate from the cellular receptors and traffic through the endocytic pathway. [4] Low pH of the endosomes and other cellular factors trigger a membrane-fusion process between viral and cellular membranes. [5] Viruses are uncoated and viral genome and proteins are released into the cytoplasm. [6] Viral RNA (vRNA) is transcribed by the RNA-dependent RNA polymerase (RdRp) and [7] mRNA is subsequently translated into different viral proteins, which are necessary to hijack the host cell machinery. [8] vRNA is synthesized and [9] new viral particles are assembled at the [9a] Golgi-complex (OWHVs, e.g., PUUV) or at the [9b] cell membrane (NWHVs). [10] Viral particles are released by fusion of the Golgi-complex (OWHVs, e.g., PUUV) or viral vesicle (NWHVs) with host cell membrane. E.E., early endosome; L.E., late endosome. Created with BioRender.com.

The two envelope glycoproteins Gn/Gc are the only viral proteins that are exposed on the virus surface and are essential for attachment to and entry into host cells (**Figure 3**). After attachment to its respective cellular receptor, the virus is internalized into the host cell. Interestingly, OWHVs, such as PUUV, enter the target cells via clathrin-dependent receptor-mediated endocytosis (111, 112), whereas NWHVs use clathrin-independent mechanisms (104, 113), such as macropinocytosis (114) or cholesterol-mediated micropinocytosis (103). Upon entry, viral particles are transported from early endosomes to late endosomal compartments. During endosomal maturation, the intra-luminal pH changes, from mildly acidic (early endosome) to strong acidic (endolysosome). This acidification process is required by the virus to detach from the bound integrin receptor and to undergo fusion of the viral with the endosomal membrane, which is mediated by a conformational change within the Gc glycoprotein (92). This fusion process consequently leads to an uncoating (112) of the virion. The hantavirus genome in form of ribonucleoproteins (RNPs) is released into the cytoplasm (115) and transcribed into S, M and L mRNAs, which are subsequently translated into proteins that are essential to hijack the host cell machinery. S and L mRNAs are translated via episomal ribosomes, whereas M-specific mRNA is translated into a glycoprotein precursor (GPC) at the rough endoplasmic reticulum (27). GPC is co-translational cleaved into Gn and Gc, most likely by host cell derived signal peptidases that are located in the lumen of the ER (116). It is thought that the cleavage site is located downstream of the conserved WAASA amino acid motif (90, 117). Shortly after the initial transcription of viral mRNA, synthesis of complementary RNA (cRNA) occurs, which serve as a template to synthesize viral RNA (vRNA) (118, 119). Both processes, transcription and replication, are mediated by the RdRp. The N-terminal part of the RdRp harbors an endonuclease activity, which allows for the cleavage and utilization of capped primers from host cell mRNAs to synthesize the viral mRNA (cap-snatching) (119). Once replication and amplification of viral genome is completed, vRNA is subsequently encapsulated by the nucleoprotein (120) and assembly of viral particles either occurs at the Golgi complex (OWHVs) (121) or at the plasma membrane (NWHVs) (122). It is assumed that the newly assembled virions bud into the Golgi complex, are transported to the cell membrane and released via exocytosis (OWHVs) (123). When assembly occurs at the plasma membrane, it is thought that viral vesicles and cell membrane fuse, and virions are released (NWHVs) (123).

### 4.5 Evasion of the human innate immune system

**Figure 4:**
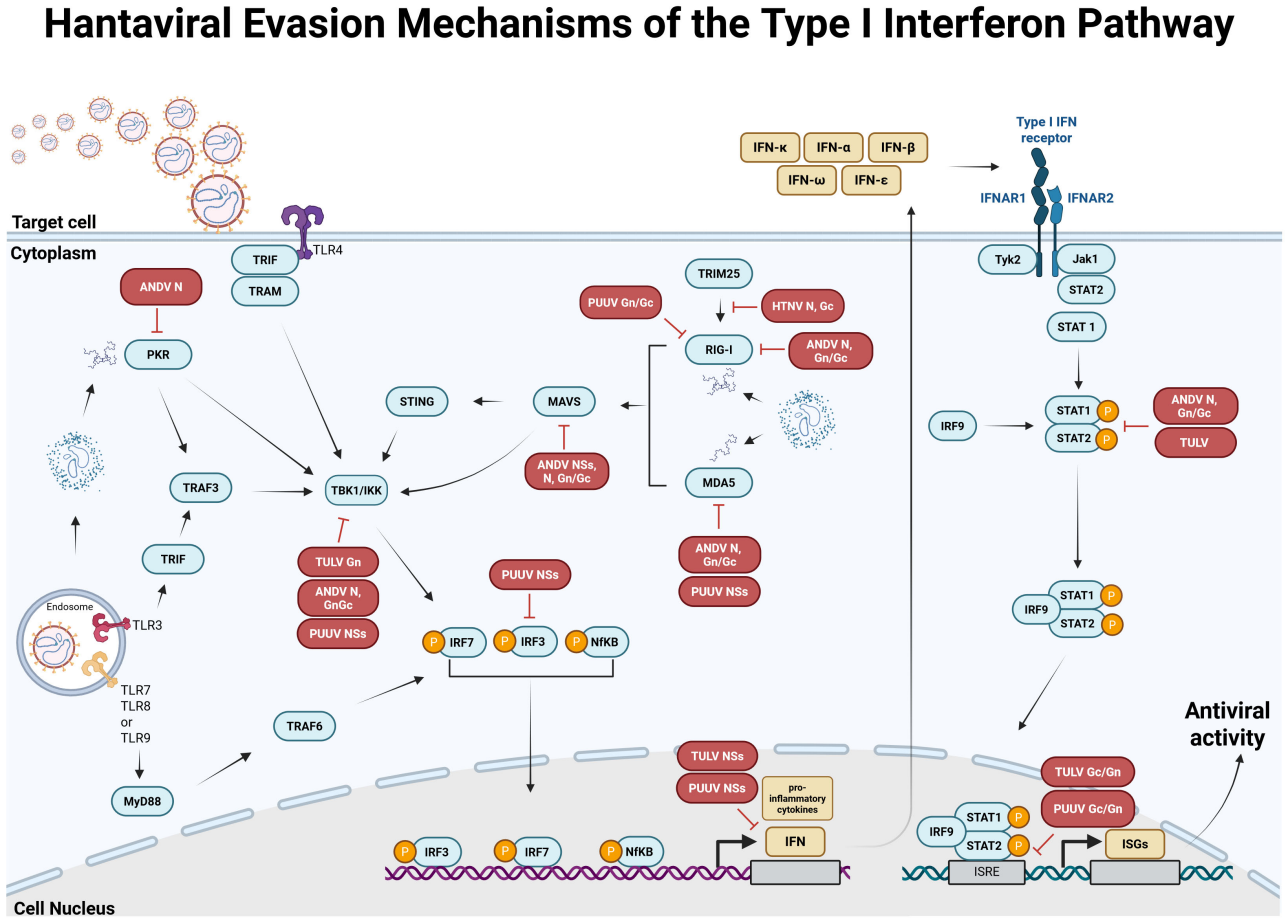
Antiviral type I interferon (IFN) response pathway and known evasion mechanisms of orthohantaviruses. Based on data from Hantaan orthohantavirus (HTNV) (127), Puumala orthohantavirus (PUUV) (128–131), Tula orthohantavirus (TULV) (128, 129, 131, 132) and Andes orthohantavirus (ANDV) (133–136). In infected cells, viral components or by-products, called pathogen-associated molecular patterns (PAMPs) are recognized by pathogen recognition receptors (PRRs), such as melanoma differentiation-associated gene 5 helicase (MDA-5), retinoic acid-inducible gene I helicase (RIG-I) or Toll-like receptors (TLRs). Receptor-ligand binding activates the type I IFN pathway resulting in the activation of TANK-binding kinase 1 (TBK1) and IkappaB kinase (IKK), which causes the phosphorylation and activation of IFN regulatory factors (IRF) 3/IRF7 and/or NfκB, leading to the expression of different type I IFNs. IFNs are released and bind to type I IFN receptors, thereby activating Janus kinase 1 (Jak1) and tyrosine kinase 2 (Tyk2). Once activated, the two proteins activate signal transducers and activators of transcription 1 and 2 (STAT1 and STAT2), which become phosphorylated and form a complex with IRF9, subsequently inducing the expression of different IFN-stimulated genes (ISGs). Hantaviruses evolved different immune evasion mechanisms to avoid detection by PRRs or interfere with downstream factors of the type I IFN pathway. These antagonisms are associated with hantavirus N (127, 133, 135–137), Gc/Gn (127, 132, 136) or NSs (128, 129, 131). MAVS, mitochondrial antiviral-signaling protein; STING, stimulator of interferon genes; TRIF, TIR-domain-containing adaptor inducing IFN-β; TRAM, TRIF-related adaptor molecule; TRAF, tumor necrosis factor receptor associated factor; TRIM, tripartite motif-containing; IFNAR, interferon α/β receptor. Created with BioRender.com.

As a response to viral infection, the host innate immune system is activated to provide a first line of defense to eliminate the virus and clear the infection [for review see (124, 125)]. Recognition of pathogen-associated molecular patterns (PAMPs), which can be viral components or by-products (e.g., double-stranded RNA during replication) occurs via pattern recognition receptors (PRRs), such as retinoic acid-inducible gene I-like RNA helicases (RLHs; e.g., melanoma differentiation-associated gene 5 helicase (MDA-5) or retinoic acid-inducible gene I helicase (RIG-I)) and Toll-like receptors (TLRs). TLRs recognize pathogens in endosomal or extracellular compartments, whereas RLHs recognize viral double-stranded RNA in the cytoplasm of infected cells (126). Upon recognition of and binding to PAMPs, the receptors mediate a signal cascade resulting in the activation of TANK-binding kinase 1 (TBK1) and IkappaB kinase (IKK) to produce type I interferons (IFNs). The released IFNs bind to their respective type I interferon receptors, resulting in the activation of the Janus kinases/signal transducer and activator of transcription proteins (JAK/STAT) pathway, which causes the expression of IFN-stimulated genes (126) (**Figure 4**). Hantaviruses have evolved several strategies to evade the host´s defense mechanism (in particular the type I interferon pathway) in order to efficiently replicate and spread in the infected host. Interestingly, different hantavirus species interfere with different modulators and regulatory factors of the type I interferon pathway, which differ also within OWHV and NWHV, and are independent of their virulence in humans (summarized in **Figure 4**). *In vitro* studies demonstrated that PUUV Gn/Gc antagonizes the IFN pathway that is stimulated via activated RIG-I (128) and interfere with the activation of IFN-stimulated response elements (ISRE) (128). In addition, PUUV NSs inhibits the activation of MDA5 (128), TBK1 (128) and interferes with the activation of interferon regulatory factor 3 (IRF3) responsive promoters and IFN-β promoters (129). TULV N inhibits the activation of RIG-I (128), and so does ANDV N (137), which additionally blocks the activation of protein kinase R (PKR) (133), TBK1 (137) and the phosphorylation of STAT1/2 (134). Furthermore, HTNV N interferes with the interaction of TRIM 25 (127) and RIG-I (127), thereby inhibiting the downstream activation of RIG-I. However, studies demonstrating the capability of PUUV N to downregulate the type I interferon pathway are lacking. Contrarily, Gallo and colleagues could confirm enhanced IFN-β promoter activity driven by PUUV N (128).

## 5 Clinical presentation and pathogenesis

The clinical presentation of PUUV infection varies from subclinical, mild, and moderate to even severe courses (138–140). The proportion of different severities is difficult to assess, as the reported numbers of PUUV infections are quite low compared to infections estimated from sero-surveillance studies (139, 141). Thus, most of the mild cases are likely missed and consequently, the clinical characteristics have been mainly derived from hospitalized patients (139). Approximately 8% of all PUUV infected patients diagnosed in a tertiary care center have been admitted to the intensive care unit (ICU) for oxygen supply (intubation and mechanical ventilation was necessary in 66%) and renal replacement therapy (applied in 66%) (142). In general, HFRS caused by DOBV is more severe with mortality rates from 5% to 15%, whereas SEOV causes moderate and PUUV and SAAV cause mild forms of disease with mortality rates of <1%. Whereas the overall mortality of PUUV infection is reported to be low, the 30-day death rate of PUUV infected patients treated at ICUs was 14% (142). The definitive reasons for the individual differences in the clinical course and outcome of PUUV infection remain unclear, but have been considered rather to be determined by factors in the human host (severe courses were associated with certain HLA alleles and genetic variation in cytokines) than by variations in PUUV virulence (143, 144). Severe PUUV infections are mainly described in male patients, but this might be explained by the exerted activities considered as risk factors for acquisition of PUUV that are more common in men (142).

**Figure 5:**
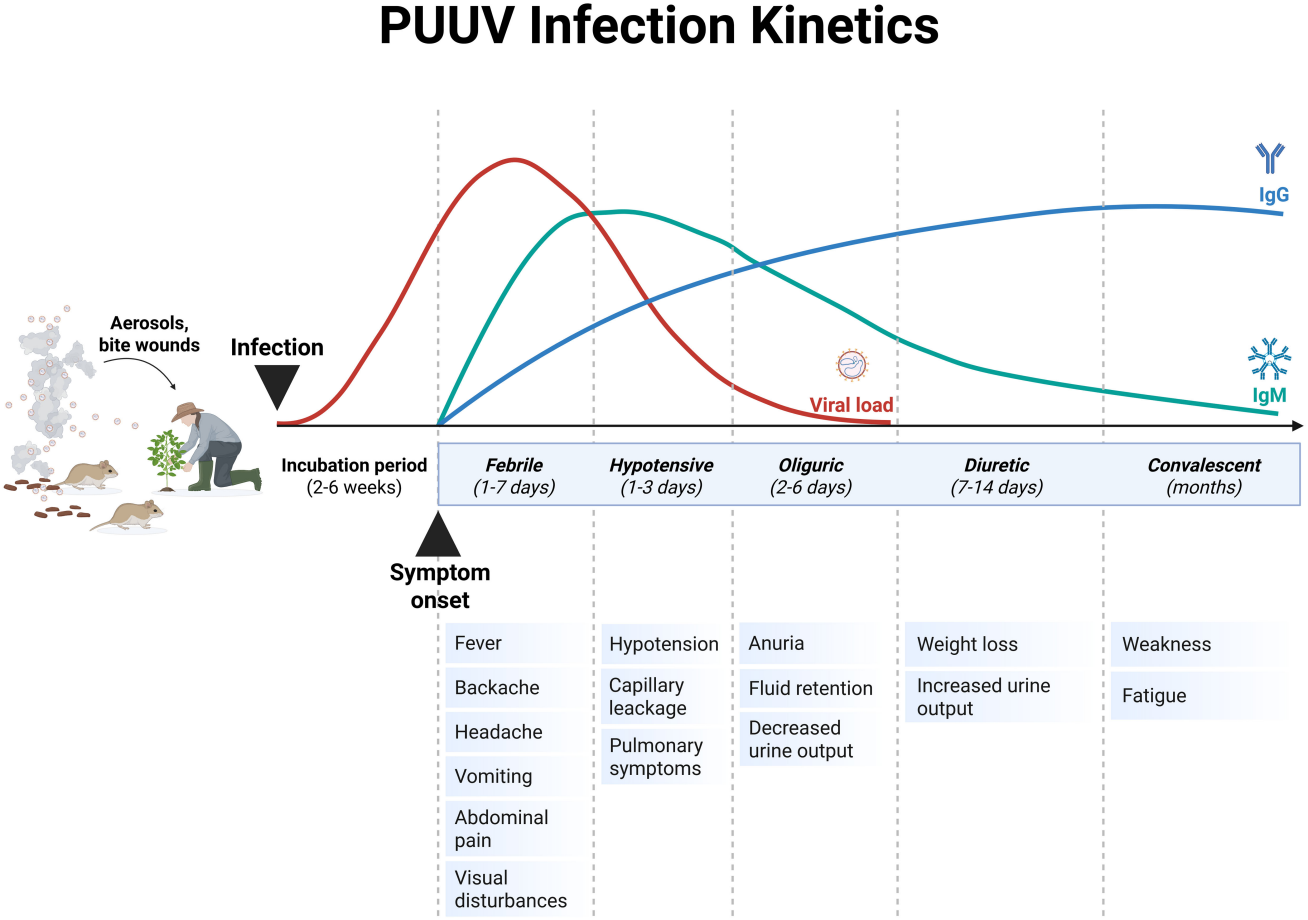
Schematic representation of the Puumala virus (PUUV) infection kinetics in humans. Typically, the severe clinical course of nephropathia epidemica (NE) that is caused by PUUV can be divided into five stages, which are not easily distinguishable: febrile, hypotensive, oliguric, diuretic and convalescent. The incubation period of PUUV infections ranges between 2-6 weeks, and is associated with an increase in viral load. The onset of the first symptoms is accompanied with an increase in antibody titers. Adapted from Avšič-Županc T et al. (138) and Mustonen et al. (145). Created with BioRender.com.

**Figure 6:**
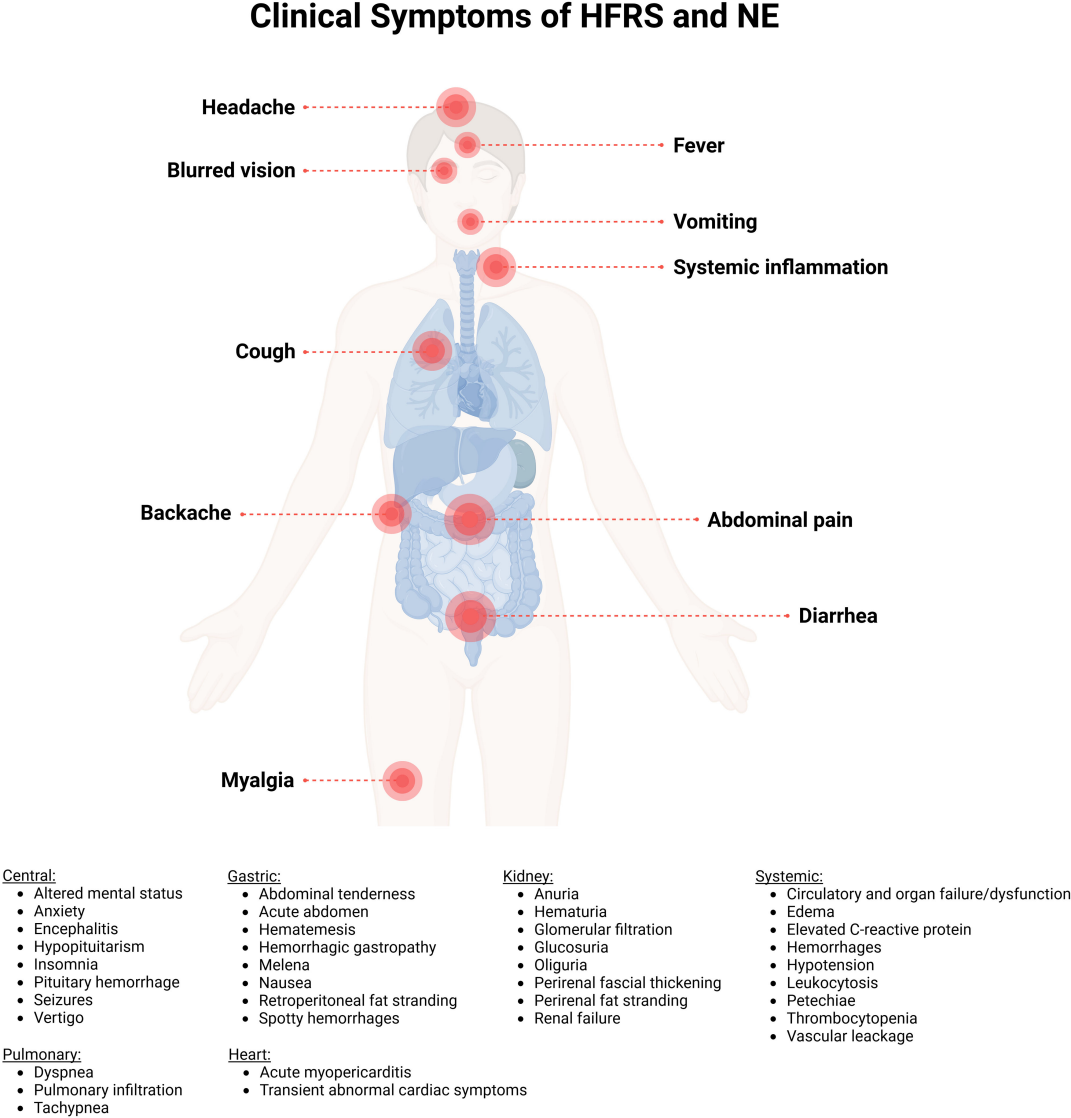
Clinical representation of hemorrhagic fever with renal syndrome (HFRS) and nephropathia epidemica (NE) caused by Puumala orthohantavirus. The main clinical symptoms are myalgia, backache, abdominal pain, vomiting, diarrhea, cough, headache, fever, systemic inflammation and blurred vision. Created with BioRender.com.

The incubation period of PUUV infection is usually 2-6 weeks (**Figure 5**), followed by unspecific symptoms and, in severe cases, organ dysfunction (139, 146). The main clinical findings of PUUV infection include fever, myalgia, headache, backache, abdominal pain, vomiting, diarrhea, cough and blurred vision (summarized in **Figure 6**) (139, 146). Vascular leakage can cause edema in many organ tissues and hypotension (139, 147). In severe cases, renal failure, marked hypotension or circulatory failure, petechiae and hemorrhages might occur (139). In case of acute kidney injury, the course of HFRS is divided into five stages (febrile, hypotensive, oliguric, diuretic, and convalescent (**Figure 5**) (138, 139). Whereas these phases are usually present in DOBV or HTNV infection, the five phases are not easily distinguishable in NE caused by PUUV (138). The urinary excretion of interleukin-6 (IL-6) correlates with the amount of proteinuria in NE (148). It has been hypothesized, that urinary IL-6 levels might reflect the production of this proinflammatory cytokine in the kidneys (147). Ultrastructural changes decrease the barrier functions of the kidney resulting in proteinuria in NE (145). Although HCPS, the disease caused by NWHV, and HFRS are separated entities, they share some common clinical characteristics. Both are characterized by the strong systemic inflammation and affection of vascular endothelial cells, leading to organ dysfunction. HCPS is characterized by respiratory symptoms, hypoxia and pulmonary infiltration in radiological examination, but HFRS can also affect the respiratory system in approximately one third to half of the patients (139, 149). In case of pulmonary involvement in PUUV infection, patients show cough, tachypnea, and dyspnea. To address the pathophysiological role of bradykinin in severe capillary leakage, the bradykinin receptor antagonist icatibant was used as treatment in some cases of severely ill NE patients (150). In ICU patients, invasive aspergillosis might complicate the course of critically ill PUUV infected patients (142). Hemophagocytic lymphohistocytosis associated with PUUV infections has also been reported and was treated with anti-inflammatory and immunosuppressive medication in one case (151, 152). A detailed description of further clinical features, findings in clinical laboratory tests and treatment are provided in the **Supplementary Material**.

## 6 Detection, diagnostics, and treatment

### 6.1 Detection and diagnostics

Several methods for PUUV detection and diagnosis have been developed. They are either based on the direct detection of PUUV genome via nucleic acid testing or on detection of antibody responses to the virus. Serological responses to virus proteins can typically already be detected at symptom onset. One of the most widely used ways to confirm PUUV infections in clinical laboratories is to measure IgM to N using enzyme-linked immunosorbent assays (ELISAs) or other immune-assays like immune-blotting or immunofluorescence assays (IFAs) and even neutralization assays (153–156).

However, nucleic acid-based detection methods have also been widely used (140, 157–160), especially in research settings and those assays may potentially detect the presence of virus genome before the onset of an antibody response (161). The challenge in diagnosing PUUV cases might often be based on insufficient awareness of physicians in areas with low or unknown PUUV prevalence, resulting in missing suspicion of PUUV infection and lack of testing. This may lead to underreporting. However, in case of availability of specific treatment options in the future (e.g., monoclonal antibodies (mAbs) or antivirals), early diagnosis may be essential to enable early treatment of patients.

### 6.2 Antivirals and immunotherapy

Currently, there are no specific targeted treatments for hantavirus infection and current strategies predominantly focus on the management of clinical symptoms. Treatment is usually symptomatic and in severe cases include oxygen supply, non-invasive or invasive mechanical ventilation, renal replacement therapy and extracorporeal membrane oxygenation (162). In individual cases, treatments with icantibant or glucocorticoids, immunoglobulins and ruxolitinib in PUUV associated hemophagocytic lymphohistocytosis have been reported (162). Several pre-clinical studies have been conducted investigating the use of both antiviral drugs and mAbs as post-exposure therapeutics.

Ribavirin (1-β-D-ribofuranosyl-1, 2, 4-triazole-3-carboxamide) is a synthetic guanosine nucleoside analog which has displayed potent broad antiviral activity against a range of RNA viruses, including hantaviruses. This antiviral activity is thought to be exerted through multiple mechanisms of action. Ribavirin has been shown to exert antiviral effects via the interruption of viral capping (163, 164) and polymerase activity (165, 166). Ribavirin also abrogates inosine monophosphate dehydrogenase (IMPDH), which results in the depletion of intracellular guanosine triphosphate (GTP) (167, 168), thereby reducing viral replication. Conversely, the antiviral effects of ribavirin against arenaviruses are independent of GTP depletion (169), and may instead be dependent on the reduction of inflammatory responses via the protection of infected cells from death (170). The inhibition of cellular GTP by ribavirin has also been shown to restrict viral infection via the induction of spermine-spermidine acetyltransferase (SSAT1) (171). SSAT1 decreases intracellular polyamine levels, which have been shown to be vital for the replication of Zika virus (ZIKV) and Chikungunya virus (CHIKV) (172). Ribavirin also acts as a mutagen, promoting the accruing of mutations in viral genomes which result in the production of defective particles and “error catastrophe” (172–175). This has been demonstrated *in vitro* to be one of the mechanisms through which ribavirin restricts HTNV infection (176), as opposed to GTP depletion (177). The protective effects of ribavirin against HTNV were first shown *in vivo* in the 1980s using a suckling mouse challenge model, wherein daily 50 mg/kg ribavirin treatment promoted survival (178). Following these promising early results, a placebo-controlled, double-blinded clinical trial was conducted using 242 HFRS patients in China which demonstrated that ribavirin therapy administered within 7 days of symptom onset reduced mortality seven-fold, prevented the induction of the oliguric phase of disease, and reduced hemorrhagic manifestations (179). These findings were recapitulated in a smaller study using US Department of Defense personnel stationed in Korea, wherein ribavirin treatment was found to restrict the renal complications of HFRS (180). The protective effects of ribavirin for treating the hantavirus cardiopulmonary syndrome caused by NWHVs are less well established. Ribavirin was found to be a potent inhibitor of ANDV infection *in vitro* and *in vivo* in the Syrian golden hamster challenge model of infection (181, 182), and in the deer mouse model of SNV infection (183). Based on these findings, an open-label clinical trial of ribavirin was conducted in the US from 1993-1994, however the results were inconclusive and no differences in mortality were observed (184). A follow-up randomized, double-blinded, placebo-controlled clinical trial was conducted in 2004 where ribavirin treatment did not show improvements in 28-day survival compared to the control group (185). Unfortunately, this study recruited only 36 patients which did not allow for robust comparisons between groups (185). Additionally, intravenous ribavirin treatment was found to be ineffective at lowering viral loads in a randomized, open-label study conducted on HFRS caused by PUUV infection (186). While the predominant side-effect of ribavirin in the treatment of HTNV was limited to a reversible hemolytic anemia (178), its usage in the treatment of PUUV infection also resulted in increased incidence of hyperbilirubinemia, sinus bradycardia, and rash (186). A phase II clinical trial (NCT00868946) was set to be carried out in Germany to investigate the efficacy of ribavirin in the treatment of HFRS, however this was withdrawn due to poor patient enrollment. Several additional antiviral drugs have been tested in combination with ribavirin to improve efficacy and limit the emergence of drug-resistant viral variants. Lactoferrin, an iron-binding glycoprotein naturally secreted in milk and saliva, has been shown to exhibit broad antiviral effects. Bovine lactoferrin has been demonstrated to inhibit SEOV cell entry *in vitro*, with complete abrogation of viral replication when used in combination with ribavirin (187). Lactoferrin has also been shown to inhibit SEOV infection *in vivo* in the suckling mouse challenge model, wherein pre-treatment with 160 mg/kg 48- and 24-hours prior to infection promoted a survival rate of 94% (188). While these results are encouraging, it is unclear how efficacious post-exposure treatment with lactoferrin would be, especially considering that the timing of therapeutic intervention is critical in the treatment of HFRS and HCPS.

Favipiravir (T-705), like ribavirin, is a synthetic nucleotide analogue which inhibits viral RNA-dependent RNA polymerases (189), abrogates viral RNA transcription (189), and promotes lethal mutagenesis (190). Favipiravir has been demonstrated to lower viral loads and promote survival in the ANDV Syrian golden hamster challenge model and to limit viral replication of hamster-adapted SNV (191). *In vitro* studies have shown that favipiravir potently inhibits HTNV and works synergistically in combination with ribavirin (192), and that it is also effective against DOBV and Maporal virus (MPRLV) (193), a NWHV which is closely related to ANDV. The synergistic effects of combination therapies with ribavirin and favipiravir have been demonstrated *in vivo* and *in vitro* for other hemorrhagic fever viruses such as filoviruses, arenaviruses (194, 195), and bunyaviruses (196, 197). While these preclinical results are promising, clinical trials are required to determine if favipiravir, either alone or in combination with ribavirin, is effective in the treatments of HFRS and HCPS including PUUV infections. An additional nucleoside analogue, 1-β-d-ribofuranosyl-3-ethynyl-[1,2,4]triazole (ETAR), has also been demonstrated to possess antiviral activity against hantaviruses. Like ribavirin, ETAR reduces intracellular GTP pools and a single study has shown that it effectively inhibits HTNV and ANDV *in vitro* and conferred improved survival in a suckling mouse HTNV challenge model (198).

Hantaviruses, as members of the *Bunyavirales* family, share some aspects of their biology with other negative sense, segmented viruses, such as the *Orthomyxoviridae*. As such, some antiviral drugs which have been characterized for the treatment of influenza virus may exhibit activity against hantaviruses. Like the *Orthomyxoviridae*, hantaviruses rely on cap-snatching for viral transcription, with the RdRp acting as a cap-dependent endonuclease (CEN) (119). CENs represent attractive targets for antiviral drug design, and a wealth of compounds have been identified for the treatment of influenza virus infection. In one study, the authors screened a library of CEN inhibitory compounds and identified several drugs which were potently antiviral against a number of bunyaviruses *in vitro* and *in vivo* (199). Of these drugs, two candidates displayed antiviral effects against TPMV, an OWHV which is apathogenic in humans, though to a lesser degree than other bunyaviruses tested (199). The authors speculate that this is due to differences in the cap snatching machinery employed by these viruses, which necessitates further study for effective hantavirus CEN inhibitors. In a separate study, one such CEN inhibitor, baloxavir acid (BXA), was found to inhibit HTNV *in vitro* and had comparable activity to favipiravir (200).

An alternative strategy for the treatment of hantavirus infection is the targeting of host proteins which are required by the virus for replication and pathogenesis. Using a small interfering RNA (siRNA) screen approach, one study identified several pro viral host proteins which promote the replication of influenza A virus (IAV). By selecting known, approved, drugs that target these proteins, the authors identified the urea-based kinase inhibitors (UBKIs) regorafenib and sorafenib as potent antiviral agents against IAV (201). These compounds also exhibited robust activity against HTNV *in vitro*, possibly via the interruption of the early stages of viral replication. In another study, authors identified a compound, 8G1, as possessing anti-HTNV activity by screening a library of kinase-inhibitors. Like regorafenib and sorafenib, 8G1 was found to inhibit the early stages of viral infection *in vitro* and effectively reduced intracellular N protein levels when administered 2-12 hours after infection (202). Using similar techniques, the same authors have further identified N6, a coumarin derivative, which inhibited HTNV replication *in vitro*, reduced organ viral titers *in vivo*, and moderately improved weight loss and survival in a suckling mice challenge model (203).

Another strategy through which host proteins can be targeted to alleviate the symptoms of HCPS is via the targeting of vascular endothelial growth factor (VEGF). It has been shown that pathogenic hantaviruses modulate the expression of VEGF as a strategy to enhance lung endothelial vascular permeability (204). Once activated, the VEGF receptor (VEGFR2) promotes the internalization and subsequent degradation of VE-cadherin, an endothelial cell junction protein which is responsible for maintaining vascular barrier function (205, 206), via a signaling pathway mediated by Src family kinases (SFKs) (207). To combat this loss of barrier function, one study utilized a panel of FDA-approved VEGFR2 and SFK inhibitors which identified several drugs that inhibited the cell permeability induced by ANDV infection *in vitro* (208). An additional study utilizing the ANDV Syrian golden hamster challenge model showed that vandetanib, a VEGFR2 antagonist, delayed the onset of severe disease, increased survival, and decreased the accumulation of fluid in the lungs (209). While these results are encouraging, there was only a moderate decrease in lung, heart, and blood virus titers three days post infection which then increased to comparable levels with control treated animals, and treatment with high doses resulted in severe side-effects (209).

An alternative to antiviral drugs is the use of neutralizing antibodies for the treatment of hantavirus infection. Early studies demonstrated that the passive transfer of sera from rabbits (20), ducks (210), rhesus macaques (211), and geese (212) vaccinated using DNA vaccine technology was protective in the ANDV Syrian golden hamster challenge model. Polyclonal alpaca IgG has also been generated via the DNA vaccination of alpacas (213). Camelid-derived IgG has the advantage of its small size which, as it is composed of only heavy chains with no light chains, allows enhanced binding to epitopes usually inaccessible to human IgG (214).

More recent studies have utilized transchromosomic cattle, in which the bovine immunoglobulin G (IgG) locus has been replaced with the human locus, for the production of anti-hantavirus polyclonal sera (215, 216). In one study, transchromosomic cattle were immunized four times using DNA vaccines encoding the M segment of either PUUV or HTNV, plasma was then drawn from the cattle, and the anti-hantavirus human IgG was purified (215). This purified polyclonal IgG was found to be potently neutralizing against HTNV and PUUV *in vivo*, protective against HTNV infection in the Syrian golden hamster challenge model, and limited infection in a marmoset model of HTNV infection (215). Similar results were achieved when the vaccination protocol was modified to focus on ANDV and SNV, resulting in polyclonal IgG which could protect from HCPS (217). In a follow- up study, transchromosomal cattle were vaccinated five times with either ANDV and SNV or HTNV and PUUV DNA vaccines prior to plasma harvest and IgG purification. The resulting polyclonal IgG exhibit strong neutralizing activity against HTNV, PUUV, ANDV, SNV, SEOV, DOBV, and Choclovirus (CHOV) and protected against infection with HTNV, PUUV and SNV *in vivo* (216). The use of transchromosomal cattle is advantageous as it allows for the rapid production of potently neutralizing human IgG in large volumes that, as it is polyclonal, targets multiple epitopes on the hantavirus glycoproteins.

MAbs have also been investigated as a potential therapeutic avenue for the treatment of hantavirus infections. Early experiments with recombinantly produced murine antibodies identified using hybridoma technology showed that neutralizing mAbs targeting the HTNV glycoprotein could promote protection from infection in suckling mice (218, 219). These studies indicated that mAbs targeting either the Gn or Gc domain of the HTNV glycoprotein were sufficient to protect from infection, a finding which was later recapitulated using hybridoma-derived mAbs obtained from mice vaccinated against ANDV (220). More recent studies have focused on the production of recombinantly produced human monoclonal antibodies derived from survivors of hantavirus infection. By screening B cells from ANDV patients, one study identified two mAbs that exhibited strong neutralization against ANDV and protected hamsters from infection when used individually and in combination (221). Strikingly, these mAbs, when administered together, provided 50% protection even when given at a later stage of infection (8- and 10-days post-infection) (222). MAbs derived from HCPS survivors have also exhibited broad activity against a range of hantaviruses, with mAbs cloned from SNV survivors showing broader neutralization than those from ANDV survivors (88). Indeed, mAbs derived from PUUV patients have exhibited exceptional cross-neutralizing activity against both OWHVs and NWHVs and represent promising therapeutics when used alone or in combination with other mAbs (85). However, so far clinical development of these mAbs has not been initiated.

## 7 Animal models

Despite ongoing research on hantaviruses since their first emergence decades ago, suitable animal models that closely mimic human HFRS or HCPS disease outcomes are still lacking for the vast majority of hantaviruses. Indeed, there are few animal models available to study different aspects of viral pathogenicity, e.g., a lethal Syrian golden hamster model for ANDV infection (223) or lethal mouse models for HTNV infection (224, 225). Even though the clinical outcome in these models do not closely recapitulate human HFRS and HCPS, parameters of a persistent or acute infection, including high viral loads and the presence of viral genome in different organs (e.g., spleen, kidney, lungs, brain), as well as seroconversion can be observed [for review (226–228)]. Nevertheless, animal models that resemble human disease outcomes more faithfully are urgently needed to investigate the efficacy of vaccines and antivirals as well as the pathogenicity of hantavirus infection in humans.

Preclinical studies on PUUV infections are typically performed in rodents and other small animal models. However, neither mice (66, 68, 69, 229–233) nor hamsters (21, 234, 235) develop clinical symptoms after PUUV infection, which is a limitation of these animal models. Nevertheless, they seroconvert after infection, and viral loads or viral antigens can be detected. Larger animals, such as non-human primates (236–238) (NHPs; e.g., cynomolgus macaques), develop clinical symptoms after PUUV infection, which partially resemble disease outcome in humans. However, due to the limited access to NHPs, their high costs and ethical reasons, they will most likely not become the standard model to study PUUV disease outcome in humans. Novel approaches besides immunocompetent small animals and non-human primates are currently being tested for both NWHVs and OWHVs. This includes animal models, in which hantavirus host receptors are artificially introduced into the animals, immunodeficient mice (239) or humanized/xenografted animal models (240–242). Those animals are more susceptible to hantavirus infection, develop clinical symptoms after hantavirus infection and partially resemble clinical signs observed in humans.

In this part, we aim to provide an overview on current animal models for hantaviruses with the focus on PUUV (summarized in **Table 4**).

**Table 4:** Current animal models to evaluate PUUV pathogenesis.

Animal model	Virus strain	Virus dose	Route of infection	% Lethality	Days until death (due to disease)	Final day	Date of last publication	Key observations	Reference
Bank voles	PUUV	n/a (wild-trapped animals)	–	0	n/a	–	1980	• PUUV antigen was detectable in the lung	(31)
	PUUV strain *Hällnäs*	10^3.5^ ID_50_ 750 ID_50_	IM IC	0	n/a	270 p.i.	1985	• Infectious virus was detectable in oropharyngeal secretions (14-28 days p.i.) and feces (35-130 days p.i.) • Viral antigen was detected in lung tissue, liver, spleen, pancreas and small intestine • No signs of infection or histopathological changes	(66)
	PUUV strain *Kazan-wt*	200 bank vole ID_50_	SC	0	n/a	133 p.i.	2008	• Seroconversion in 10/10 infected animals (IgG positive) • Detection of viral RNA in urine (14-28 days p.i.), saliva (11-28 p.i.) and feces (11-28 days p.i.) • Detection of viral RNA in 5/6 animals at day 133 p.i. • Intranasal infection of naïve animals with infectious urine, feces or saliva resulted in seroconversion (7/14 animals)	(69)
	PUUV-strain *Suonenjoki* (PUUV-*Suo*), PUUV strain *Kazan*, PUUV-wt	10,000 FFFU	SC	0	n/a	35 p.i.	2020	PUUV-*Suo*: • Viral RNA was detected in lungs, spleen and kidneys (3 – 35 days p.i.) and urine (3-14 days p.i.) • Detection of PUUV-specific Ig at 14 days p.i., which decreased until day 35 p.i. • No histopathological changes • PUUV N antigen was found in pneumocytes, macrophages and capillary endothelial cells (3 days p.i.). • PUUV N antigen was found in macrophages of splenic red pulp (14-35 days p.i.) PUUV strain *Kazan*: • Detection of viral RNA in lungs (3-7 days p.i.), spleen and kidneys (3 days p.i.) • No histopathological changes • No PUUV N antigen was detected in organs • Detection of PUUV-specific Ig at 7 days p.i., which decreased until 14 days p.i. and were not detectable after 21-35 days p.i. PUUV-wt: • Detection of viral RNA in lungs, spleen and kidneys (35 days p.i.) • No histopathological changes • Detection of PUUV N antigen in lungs, kidneys and spleen in 1/4 animals • Detection of PUUV-specific Ig at 21 days p.i. in 1/3 animals, which remained stable until day 35 p.i.	(68)
	PUUV	n/a (wild-trapped animals)	–	0	n/a	–	2023	• Seroconversion in 36/188 wild-trapped animals • PUUV N antigen was detectable in liver, stomach, kidney and testis • No histological findings in sampled organs	(62)
Syrian golden hamsters	PUUV strain *Sotkamo*	3,300 FFU	SC	0	n/a	70 p.i.	2011	• No clinical symptoms • Seroconversion: o small differences in IgG response between 4- and 8-week-old animals o more IgM response in 4-week-old animals o no differences in nAb response between 4- and 8-week-old animals • Viral RNA was detected in various organs (lung, kidney, spleen, liver, heart), higher viral load in 4-week-old animals • Viral N antigen was detected in lung samples of 4-week-old animals, but not in 8-week-old animals • Slight inflammatory reactions in lung adrenal gland, cerebellum of 4-week-old animals • 4-week-old hamster showed persistent infection	(235)
	PUUV strain *Sotkamo*	1,000 or 10,000 PFU + re-exposure with 200 PFU ANDV	Oral IM	0	n/a	10-28 post re-exposure	2017	• PUUV survives in gastric fluid (pH 3: <10 min, pH: 4-7 < 15min) Pre-exposure: • Seroconversion in 2/8 (1,000 PFU) and 3/8 (10,000) infected animals Re-exposure (day 35 post pre-exposure): • 5/5 ANDV infected animals survived	(21)
	PUUV strains *Beaumont, Seloignes*	1,000 PFU (low dose model)	IM IN	0	n/a	28 p.i.	2019	• Seroconversion started at day 24 p.i. • Viral genome was detectable between days 11-17 for IM infected animals in brain, kidney, liver, heart • Viral genome was only detectable at day 28 in the brain of IN infected animals • No infectious virus was detectable in heart, liver, lung, spleen, kidney, brain, serum or urine in neither IM nor IN infected animals • No changes in white blood cell or platelet numbers during infection	(234)
Neutered and descented ferrets (*Mustela putorius furo*)	PUUV strains *K27, Beaumont,* *Seloignes*	pre-exposure: 2,000 PFU + re-exposure: 94,000 PFU (*Beaumont*) or 164,000 PFU (*Seloignes*)	IN (pre-exposure) IM (re-exposure)	0	n/a	50 p.i. (re-exposure)	2019	Pre-exposure: • No seroconversion was observed Re-exposure (day 35 post pre-exposure) • Weight loss started at day 3 p.i. • Seroconversion in all animals by day 35 p.i. • Neutralizing antibodies were detected by day 28 p.i. • No changes in white blood cell or platelet numbers • No viremia in serum Immunosuppression (day 42 post re-exposure): • Ferrets succumbed most likely due to secondary infection, not due to PUUV infection • Small amount of viral genome in liver and spleen • No infectious virus was found in heart, lung, liver, spleen, kidney, intestine	(234)
	PUUV strain *Beaumont*	94,000 PFU	IM	0	n/a	35 p.i.	2019	• Weight loss started at day 3 p.i. • No clinical symptoms (fever, proteinuria, hematuria) • Antibody response detectable at day 14 p.i. • Neutralizing antibody response detectable at day 28 p.i. • Pathology: no changes in cerebrum, pituitary gland, cerebellum, adrenal gland, spleen, lung, heart, liver, small intestine	(234)
Cynomolgus macaques *(Macaca fascicularis)*	PUUV strain *Hällnäs*	10^6^ TCID_50_	IT	0	n/a	6 days to 30 weeks p.i.	1995	• Clinical symptoms: apathetic, appetite loss, skin rash, slight proteinuria, microhematuria • Recover from illness, no clinical symptoms at late time points • No abnormalities in heart, lung, urinary bladder, brain, aorta, glomeruli, cortex • No changes in blood chemistry • Slight abnormalities in tubules of kidneys (medullary epithelium: pycnotic nuclei, desquamated cells) • Detection of viral antigens (N, Gn/Gc) in kidney sections • Seroconversion: PUUV-specific IgA, IgM and IgG antibodies	(236)
	PUUV strain *Kazan-wt*	10^5^ bank vole ID_50_	IV	0	n/a	28 p.i.	2002	• Clinical symptoms: appetite loss, apathetic, fever, proteinuria, polyuria • Recover from illness, no clinical signs at later time points • Elevated levels of CRP, creatinine and NO • Detection of PUUV S RNA in plasma and tissue samples (kidney, lung, liver, heart, spleen) • Seroconversion: detection of PUUV-specific IgM and IgG antibodies • Detection of neutralizing antibodies • Increased levels of plasma cytokines: IL-6, TNF-α, IFN-γ, IL-10	(237)
	PUUV strain *Kazan-wt*	10^5^ bank vole ID_50_	IV	0	n/a	28 p.i.	2008	• Clinical symptoms: apathetic, appetite loss, fever, proteinuria, hematuria, • Elevated levels of NO, CRP, creatinine • Increased levels of plasma cytokines: IL-6, TNF-α • Detection of PUUV S antigen in kidney, liver and spleen • Focal lymphocyte infiltrates in kidney, lung, heart • Detection of inflammatory cells at sites of tubular damages	(238)

IM, intramuscular; IC, intracerebral; IT, intratracheal; IN, intranasally; SC, subcutaneous; IV, intravenous; p.i., post infection; FFFU, fluorescent focus-forming units; FFU, focus forming units; PFU, plaque forming units; TCID_50_, tissue infectious dose 50; nAb, neutralizing antibodies; ANDV, Andes orthohantavirus; PUUV, Puumala orthohantavirus; n/a, not applicable.

### 7.1 Rodent models

Rodents are the natural reservoir of many of the hantaviruses, in which the viruses mainly cause a persistent infection. Investigations on disease outcome and the presence of viral RNA or infectious virus are either done by using wild-trapped animals or animals, which are infected in a controlled laboratory setting. Experimental infection of bank voles with PUUV resulted in seroconversion. Furthermore, viral RNA and infectious virus could be detected in serum, lung, spleen, kidneys, urine, feces and saliva; however, the animals did not succumb to the infection (66, 68, 69). No specific histological findings or signs of infection were observed in wild-trapped or experimentally infected bank voles, and PUUV N antigen was detectable in several organs, including kidney, lung, testis, liver and stomach, indicating a broad organ tropism (31, 62, 66, 68). Studies on hantavirus infections using rodent models demonstrated a strong correlation between the age of rodents and the disease outcome. Infection of 3-day-old suckling mice (*Mus musculus*) with HTNV caused 100% lethality, only 50% lethality in 1-week-old mice and no lethality in 2-week-old mice (229). Similar effects were observed upon infection of newborn rats with HTNV (230). Commonly used laboratory mouse strains (BALB/c, C57BL/6, SJL/J) have been susceptible to HTNV infection, however, mice had to be infected intraperitoneally (IP) with high doses, which do not mimic the natural route of infection in humans. Additionally, infected animals died due to acute encephalitis, which is not a typical symptom of HFRS (231). Due to the short time window in which rodents are susceptible to infection and the differences in the clinical outcome compared to human hantavirus infections, these models are not suitable to investigate the efficacy of antivirals and the protective capacity of vaccine candidates for translation to humans. Immunodeficient mice, such as Nlrc3^-/-^ mice were more susceptible to IP infection with HTNV compared to C57BL/6 wild-type mice (232), indicated by higher weight loss and higher viral load in different organs (e.g., spleen, kidney). Humanized mice, such as hNSG/HLA-A2 mice (240) were highly susceptible to infection with HTNV. Mice showed weight loss, ruffled fur, decreased activity and inflammatory activities in the lung tissue. Furthermore, these animals showed reduced numbers of platelets in the blood, which has been also observed in hantavirus-infected humans (233).

### 7.2 Syrian golden hamster

Studies have shown that Syrian golden hamsters do not develop clinical symptoms upon intramuscular (IM) (234), subcutaneous (SC) (235), or intranasal (IN) (234) infection with PUUV. In addition, no changes in white blood cell numbers or platelet numbers, which are associated with leukocytosis and thrombocytopenia in human HFRS patients (243), respectively, were observed. However, animals seroconverted and viral genome could be detected in various organs, such as brain, kidney, liver and heart of IM (21) and SC (235) infected animals. In contrast, hamsters that were IN (234) infected showed only detectable amounts of viral genome in the brain, but not in other organs. Witkowski and colleagues (21) could demonstrate seroconversion in hamsters that were experimentally infected with PUUV via the gastric route, which subsequently provided protection against lethal ANDV infection. Sanada and colleagues (235) demonstrated an age-dependent effect on PUUV persistence. Four-week-old hamsters showed persistence up to 70 days post infection, higher viral load in various organs compared to 8-week-old hamster, and slightly increased inflammatory responses in lung, adrenal gland and cerebellum. Moreover, viral N antigen was detectable in 4-week-old hamster, but not in 8-week-old hamster.

### 7.3 Ferrets

Studies have shown, that ferrets do not develop clinical symptoms except for weight loss upon infection with PUUV (234). In addition, no pathological changes were observed in different organs, including lung, heart, liver, cerebrum or small intestine. However, seroconversion could be confirmed, including the detection of neutralizing antibodies (234).

### 7.4 Cynomolgus macaques

Cynomolgus macaques are more susceptible to PUUV infection compared to other animals, however, they do not succumb the infection (236–238). Intratracheal (IT) (236) or intravenous (IV) (237, 238) inoculation with PUUV led to the development of clinical symptoms (appetite loss, apathy, skin rash, proteinuria, microhematuria, fever, polyuria). However, the animals fully recovered after a few days. No abnormalities were observed in various organs, such as the liver, brain, heart, or urinary bladder, but slight abnormalities were seen in the tubules of the kidneys, which were limited to the medullary epithelium. Viral antigens could be detected in kidney sections and other tissues, such as lung, liver, heart or spleen (237, 238). Seroconversion was confirmed by detecting PUUV-specific IgG, IgA, IgM and neutralizing antibodies (237). Increased levels of plasma cytokines (IL-6, TNF-α, IFN-γ, IL-10) could be detected (237), which is typically found in human NE patients (244). Inflammatory cells were detected at sites of tubular damage, indicating that PUUV replication provokes immunopathology induced by activated T cells. Furthermore, the authors observed a correlation between high viral load and disease severity (238).

## 8 Vaccine approaches

Multiple vaccine candidates to prevent HFRS, mainly targeting HTNV or SEOV (245), have been developed using inactivated virus grown in cell culture or rodent brains, and were evaluated in preclinical and clinical trials in Asia. However, none of them were approved for human use in the US or Europe, mainly because of the used vaccine platforms employed and the targeted hantavirus species. Due to safety concerns, rodent brain-derived vaccines are no longer suitable for use in humans (246). In addition, there is only little cross-reactivity among certain hantavirus species (247), and as PUUV is the primary circulating hantavirus species in Europe, vaccines based on HTNV or SEOV would not be effective in Europe.

The very first candidate vaccine for prevention of HFRS, Hantavax, was already developed in 1988 by Lee and colleagues (248), by propagating the Hantaan virus ROK 84-105 strain on suckling mouse brains, followed by an inactivation step with 0.05% formalin. By demonstrating seroconversion with both ELISA and IFA as a surrogate for the efficacy of the vaccine, Hantavax was approved in 1990 in Korea for human use. However, the premise was to demonstrate in the subsequent years the protective efficacy of Hantavax in a controlled clinical trial compared to a placebo control group, and to demonstrate long-term maintenance of protection (249). The recommended vaccination schedule was a primary immunization with two doses one month apart, followed by a booster immunization one year later (0-1-13 schedule). However, by 2018 the Ministry of Food and Drug Safety of Korea changed this recommendation from three to four immunizations (250). Since its licensing more than 30 years ago, several million doses of Hantavax were administrated (251). However, its effectiveness, which is primarily determined by measuring humoral immune responses as a correlate of protection, is still debated (252, 253). Clinical trials with Hantavax had demonstrated a need for optimization for both, the recommended doses and immunization schedule, as the rate of seroconversion in the vaccinees was low, followed by a swift decline in titers of neutralizing antibodies (254–256). Song and colleagues (254) performed a phase III, multi-center clinical trial by immunizing healthy adults with Hantavax according to the recommended 0-1-13 immunization schedule. One month after the primary immunization with two doses, seroconversion was detected in 90% of the vaccinees via indirect IFA and in only 23% of the vaccinees via plaque-reduction serum neutralization assay (PRNT_50_). The rate of seroconversion declined to the pre-vaccination level after one year, however, the booster immunization led to an increase of the seroconversion rate by 87% (IFA) and 45.07% (PRNT_50_). Based on these observations, Song and colleagues (255) performed an additional multi-center phase III clinical trial immunizing healthy adults with Hantavax using a modified immunization schedule with three doses for primary vaccination followed by a booster immunization one year later (0–1–2–13 schedule). One month after the third primary vaccination, the seroconversion rate was 92.81% (IFA) and 80.97% (PRNT_50_) and declined to almost pre-vaccination level before the booster immunization. One month after the booster vaccination, seroconversion was detectable in 96% (IFA) and 67% (PRNT_50_) of the vaccinees. However, it decreased to around 40% a few months later.

Over the last years, several new strategies, such as virus-like particles (VLP) vaccines, recombinant protein vaccines, subunit vaccines, recombinant viral-vector vaccines, and nucleic acid-based vaccines, were developed and served to generate vaccine candidates, mainly targeting ANDV, DOBV, HTNV or PUUV [for review (257, 258)]. In this part, we aim to provide detailed information about the current status of preclinical (summarized in **Table 5**) and clinical testing (summarized in **Table 6**) of vaccines targeting PUUV. The main targets for HFRS vaccine research are Gn/Gc and N. Gn/Gc was found to induce high levels of neutralizing antibodies (280), which are thought to be the main correlate of protection against hantavirus infection (281). N is thought to induce mainly cellular immune responses (265, 280), and although N-specific antibodies are induced upon immunization, they show poor neutralizing activity (264, 274). However, N has the advantage of inducing immunogenicity independent from post-translational modifications, which allows for an efficient production in cost-effective expression systems, such as *Escherichia (E.) coli*. Furthermore, the amino acid sequence of N among hantavirus serotypes is more conserved compared to Gn/Gc, thus, N might be a good target to generate cross-protective vaccines (282).

**Table 5:** PUUV vaccine candidates evaluated in different animal models.

Vaccine platform	Antigen/Virus	Model	Route of administration	Date of last publication	Key observations	Reference
Inactivated virus vaccine	Puumala (strain *PUUTKD/VERO*), Hantaan (strain *HTN-P88/VERO*), Sochi (strain DOB-*SOCHI/VERO*)	BALB/c mice	IM	2020	• Balanced immune responses against PUUV, HTNV and SOCHIV • No differences in induced level of neutralizing antibodies between two or three immunizations • Vaccines can be stored up to 2 years and still induce robust immune responses	(259)
	Puumala (strain *PUU-TKD/VERO*) + adjuvants (SP, Al, LTB, LPS)	BALB/c mice	IM	2020	• Induction of a substantial number of neutralizing antibodies in all groups • LPS (50 µg/ml), SP (300 µg/ml), LTB (0.2 µg/ml) significantly enhanced immune responses after two and three immunizations compared to non-adjuvanted vaccine preparations	(260)
Recombinant protein vaccine	PUUV N (strain *Sotkamo*)	Bank voles	n/a	1996	• Immunization with full-length rN, truncated rN_1-79_, rN_1-118_, rN_229-327_, rN_1-267_, or synthetic N_241-270_ peptide induced high levels of anti-PUUV N-specific IgG antibodies • No neutralizing antibodies were detected, besides after immunization with rN_1-267_ (low level) • Immunization with full-length rN, and truncated rN_1-79_, rN_1-118,_ rN_1-267_ protected voles from PUUV infection • Immunization with rN_229-327_ partially protected voles from infection with PUUV (1/3 animals positive for PUUV N antigen)	(261)
	PUUV N (strain *Kazan-E6)*	BALB/c, CBA, C57BL/6 mice	IP SC	2001	• Seroconversion in all three mouse strains (CBA>BALB/c>C57BL/6) • IgG subclasses IgG1, IgG2a, IgG2b were detectable 2 and 4 weeks after immunization in all three mouse strains, whereas IgG3 was only detectable in C57BL/6 • Antigenic regions within PUUV N were identified and are located within the N-terminal part of the protein (aa 1-120) and C-terminal part (aa 396-420; CBA mice) • Th-cell immunogenicity in all three mouse strains (CBA>BALB/c>C57BL/6) • Cytokine release (*in vitro* stimulation with PUUV N) of lymph node lymphocytes confirmed for all three mouse strains (IFN-γ, IL-2) or only BALB/c (IL-4) and C57BL/6 (IL-6)	(262)
	PUUV N	New Zealand white rabbits	IM IP	2001	• Induction of PUUV N-specific antibodies upon immunization	(263)
	PUUV N (*PUUV/Kazan*), TOPV N (*TOPV/Ls136V5/94*), DOBV N (*strain DOBV/Slovenia L41916)*, ANDV N ANDV (AF004660)	Bank voles	IM	2002	• Immunization with PUUV rN and TOPV rN protected against PUUV infection • Immunization with DOBV rN and ANDV rN protected partially against PUUV infection • Highest cross-reactivity against PUUV N antigen in ANDV > TOPV > DOBV	(264)
	PUUV N (strain *Vranica/Hällnäs*)	Bank voles	n/a	2002	• Immunization with authentic PUUV N led to partial protection against PUUV • Immunization with his-tagged PUUV N emulsified in Freund’s adjuvant led to complete protection against PUUV • Immunization with his-tagged PUUV N emulsified in alum led to partial protection against PUUV	(265)
	PUUV N	BALB/c mice	oral IP	2004	• No differences in PUUV N-specific antibodies between vaccinated and control group • Booster immunization (IP) after ten weeks did not induce memory immune responses	(266)
	PUUV N	NMRI mice	SC	2008	• Three immunizations with high doses (10 µg) of Puu-N (full-length N), P40-Puu-N (full-length N bound to rP40), or P40-Puu 118 (truncated N_1-118_ bound to P40) elicited high PUUV N-specific antibody responses • All mice immunized three times with 10 µg P40-Puu 118 were fully protected against PUUV infection and partially with 10 µg Puu-N or P40-Puu-N • Partial or no protection against PUUV infection with low dose (0.2 µg) or medium dose (2 µg) in combination with different immunization time points for all three vaccines • Single immunization with 10 µg of all three vaccines induced CTL activation	(267)
	Chimeric multi-epitope vaccine based on PUUV G, HTNV G, SEOV G	BALB/c mice	IM	2012	• Induction of neutralizing antibodies against HTNV and SEOV • Induction of cytokines (IFN-γ, IL-4 and IL-10)	(268)
DNA vaccine	PUUV N (strain *Vranica/Hällnäs*)	BALB/c mice	IM	2000	• Detection of PUUV-N specific antibodies 6 and 11- weeks post infection • Reactivity to B cell epitopes along full-length N protein	(269)
	PUUV N (strain *Sotkamo*) + modifications (intracellular form, GPI anchored form, secreted form, TM form)	Bank voles	IM	2001	• No seroconversion and no protection against PUUV infection in voles immunized with the intracellular form • Partial seroconversion and partial protection against PUUV infection in voles immunized with the secreted, TM and GPI anchor form	(270)
	PUUV N (strain *Sotkamo*) + modifications (intracellular form, GPI anchored form, secreted form, TM form)	BALB/c mice	IM	2001	• No seroconversion in mice immunized with the intracellular form • Full seroconversion in mice immunized with the secreted form • Partial seroconversion in mice immunized with TM and GPI anchored form	(270)
	PUUV N (*Umea/hu*), SEOV N (*Sapporo SR-11*), SNV N (*Convict Creek 107*)	BALB/c (cJBom) mice	ID	2007	• High antibody titers towards corresponding N protein • Low cross-reactivity of PUUV N against SEOV and SNV • Low cross-reactivity of SEOV N against PUUV and SNV antigens • High cross-reactivity of SNV N against PUUV and moderate against SEOV antigens • Strong immunogenic region located near the amino terminus of N	(271)
	PUUV and/or HTNV Gn/Gc	Syrian golden hamster	IM ID	2008	Single PUUV DNA vaccine administration: • Induction of PUUV-specific antibodies • Poor protection against HTNV infection Combined PUUV and HTNV DNA vaccine administration: • If administered at same injection site, only antibodies against PUUV detected and poor protection against HTNV • If administered at different injections sites, antibodies against PUUV and HTNV detected and protection of all hamsters	(272, 273)
Virus-like particle vaccine	PUUV N (strain *CGlS-20*)	Bank voles	SC	1998	• High titer of N-specific antibodies in all groups • Particles carrying N_1-45_ protein provided protective efficacy in 4/5 animals • Particles carrying N_38-72_ protein provided no protective efficacy • Particles carrying N_75-119_ protein provided protective efficacy in 1/4 animals	(274)
Recombinant Lentivirus vaccine (microvesicles)	PUUV N or PUUV Gc/Gn	C57BL/6 mice	SC	2022	• Induction of PUUV-specific humoral and cellular immune responses • Activation of cytokines/interleukins (TNF-α, IFN-γ, GM-CSF, CCL11) • No differences observed in immunogenicity when using different vaccine doses (30 µg, 20 µg, 15 µg, 10 µg)	(275)
	PUUV N and/or PUUV Gc/Gn	C57BL/6 mice	SC	2024	• Immunogenic regions at the N-terminal part of N • More robust immune response with PUUV N/G • Cross-reactivity against HNTV > DOBV > ANDV • Cross-reactive peptides located at C- and N-terminal part of N	(276)

IM, intramuscular; IP, intraperitoneal; ID, intradermal; SC, subcutaneous; ANDV, Andes orthohantavirus; DOBV, Dobrava-Belgrade orthohantavirus; HTNV, Hantaan orthohantavirus; PUUV, Puumala orthohantavirus; SEOV, Seoul orthohantavirus; SNV, Sin Nombre orthohantavirus; SOCHIV, Sochi orthohantavirus; TOPV, Topograf orthohantavirus; LPS, Lipopolysaccharide; SP, spherical particle; LTB, Heat-labile enterotoxin B; (r)N, (recombinant) nucleoprotein; Gn/Gc, envelope glycoproteins; n/a, not applicable; TM, transmembrane; GPI, glycosylphosphatidylinositol; P40, outer membrane protein A; CTL, cytotoxic T cell.

**Table 6:** PUUV vaccine candidates evaluated in clinical trials.

Vaccine platform	Type of candidate vaccine	# of doses	Schedule	Route of administration	# of participants (enrolled)	Date of last publication	Phase (Trial registries, public reports)	Key observations	Reference
DNA vaccine	PUUV and/or HTNV Gn/Gc	3	Days 0, 28, 56 (0-1-2 schedule)	ID (PMED)	27	2012	Open-label, single-center phase I (NCT01502345)	• No serious severe adverse events • Seroconversion in 30% (HTNV vaccine group), 44% (PUUV vaccine group) and 56% (PUUV/HTNV group) • Decline of neutralizing antibodies by day 180	(246)
	PUUV and/or HTNV Gn/Gc	3	Days 0, 28, 56 (0-1-2 schedule)	IM (IM-EP)	27 (31)	2014	Open-label, single-center phase I (NCT01502345)	• No serious severe adverse events • Seroconversion in 56% (HTNV vaccine group), 78% (PUUV vaccine group) and 78% (PUUV/HTNV vaccine group) • Participants from the PUUV/HTNV group responded stronger to PUUV than to HTNV	(277)
	PUUV and HTNV Gn/Gc (optimized)	3-4	Days 0, 28, 56, 168 (0-1-2-6 schedule)	IM (IM-EP)	120	2020	Phase IIa randomized, double-blind (NCT02116205)	• No serious severe adverse events • Induction of high titers of neutralizing antibodies against HTNV and PUUV • Subjects immunized four times with 1 mg vaccine each elicited highest seropositivity rate • Decline of antibodies 4-5 months after last immunization • Evidence of recall response after last immunization	(278)
	PUUV and/or HTNV Gn/Gc (optimized)	4	Days 0, 28, 56, and 168 (0-1-2-6 schedule)	IM (PharmaJet Stratis^®^ needle-free injection system)	22 (27)	2024	Randomized phase I clinical trial (NCT02776761)	• No serious severe adverse events • nAb detectable in 100% (HTNV and PUUV vaccine groups) and 44% (HTNV/PUUV vaccine group) • Cross-reactivity against HTNV in PUUV vaccine group and vice versa • Cross-reactivity against DOBV detectable in all three groups • Participants from the PUUV/HTNV group responded stronger to PUUV than to HTNV • nAb responses in all three vaccine groups still detectable 6 months after the last vaccination	(279)
Inactivated virus vaccine	Formalin-inactivated suckling hamster brain-derived HTNV/PUUV combination vaccine	3	Days 0, 28, 56 (0-1-2 schedule)	SC	10	2002	n/a	• No serious severe adverse events • High levels of neutralizing antibodies against HTNV and PUUV after second and third vaccination	(251)

ID, intradermal; HTNV, Hantaan orthohantavirus; PUUV, Puumala orthohantavirus; DOBV, Dobrava-Belgrade orthohantavirus; PMED, particle-mediated epidermal delivery; IM-EP, intramuscular electroporation; SC, subcutaneous; Gn/Gc, envelope glycoprotein; nAb, neutralizing antibodies; n/a, not applicable.

### 8.1 Inactivated whole virus vaccines

Dzagurova and colleagues (259) established a polyvalent vaccine, based on β-propiolacton inactivated cell culture preparation of the Hantaan HTN-P88/VERO strain, the Puumala PUU-TKD/VERO strain and the Sochi DOB-SOCHI/VERO strain (SOCHIV), and evaluated its immunogenicity in BALB/c mice. Mice were immunized intramuscularly two to three times two weeks apart with 0.5 ml (52 µg total protein/ml) of the vaccine, either undiluted or diluted (1:2, 1:8, 1:32), and the level of induced neutralizing antibodies were determined two weeks after the last immunization. In general, the polyvalent vaccine elicited neutralizing antibodies equally to SOCHIV, PUUV, and HTNV, providing a balanced immune response. There was no difference in the level of induced neutralizing antibodies and cytokines in mouse sera (IL-1β, IL-12, IFN-γ) between two and three immunizations.

Kurashova and colleagues (260) generated an inactivated PUUV vaccine, based on the propagation of the Puumala PUU-TKD/VERO strain on Vero cells and subsequent inactivation with β-propiolacton, and tested the beneficial effect of different adjuvants (subunit of an *E. coli* derived heat-labile enterotoxin (0.2 µg/ml, 7.5 µg/ml), aluminum hydroxide (1 mg/ml), spherical particles of coat protein from tobacco mosaic virus (100 µg/ml, 150 µg/ml, 300 µg/ml, and lipopolysaccharide (low endotoxic) from *Shigella sonnei* (50 µg/ml)) upon vaccination of BALB/c mice. Mice were immunized intramuscularly three times two weeks apart with the vaccines, either undiluted or diluted (1:2, 1:4, 1:8). All vaccines induced a substantial titer of neutralizing antibodies after two and three vaccinations. Interestingly, aluminum hydroxide (283), which is commonly used as adjuvant in inactivated vaccine preparations, did not lead to an increase of induced neutralizing antibodies compared to the non-adjuvanted vaccine group. Immunization with PUUV vaccines adjuvanted with either the lipopolysaccharide, the B subunit of heat-labile enterotoxin or spherical particles (300 µg/ml) significantly increased the humoral immune responses, also when administered in a diluted formulation.

Cho and colleagues (251) reported immunogenicity data from a small clinical study with 10 participants, who received three times four weeks apart a combined PUUV/HTNV vaccine, which was developed by propagation of the viruses on suckling hamster brains with subsequent formalin inactivation. The vaccine was well tolerated and induced high levels of neutralizing antibodies against HTNV and PUUV after the second and third immunization.

### 8.2 Recombinant protein vaccines

Maes and colleagues (267) linked the outer membrane protein A of *Klebsiella pneumoniae* (rP40) to a full-length (P40-Puu-N) or a truncated (P40-Puu 118) form of PUUV N and compared their immunogenic properties to an unmodified full-length PUUV N vaccine (Puu N). Outbred NMRI mice were immunized subcutaneously (SC) once, twice or three times with different doses (0.2 µg, 2 µg, 10 µg) of the three vaccines. NMRI mice were chosen, because they have been described previously as a suitable non-lethal rodent model, as the mice readily seroconvert and show detectable levels of neutralizing antibodies after infection (284). Overall, there was a dose and frequency of immunizations depended effect on the induction of antibody responses, with three immunizations with 10 µg of each vaccine eliciting the highest responses. In addition, full protection against PUUV was only seen with three immunizations of 10 µg P40-Puu 118. All three vaccines induced substantial numbers of cytotoxic T lymphocytes (CTLs) after a single immunization with 10 µg of each vaccine.

De Carvalho Nicacio and colleagues (262) evaluated the immunogenic properties of recombinant *E. coli* expressed PUUV N in three different mouse strains (CBA, BALB/c and C57BL/6), focusing on IgG subclasses and T-helper (Th) lymphocyte responses. Mice were immunized with 20 µg via the intraperitoneal (IP) route to determine antibody responses and with 50 µg via the SC route to determine Th lymphocyte responses. Overall, seroconversion was observed in all three mouse strains, with the highest titers for CBA > BALB/c > C57BL/6. All IgG subtypes were detectable 2 and 6 weeks after immunization in all three mouse strains, besides IgG3, which was only found in small amounts in C57BL/6 mice 2 weeks after immunization. Th lymphocyte responses could be confirmed in all three mouse strains, and immunogenic epitopes were identified across PUUV N, mainly located at the N-terminal part of the protein. *In vitro* stimulation of Th lymphocytes with PUUV N induced the release of different cytokines, including IFN-γ and IL-2 (all three mouse strains), as well as IL-4 (CBA) and IL-6 (C57BL/6).

Lindkvist and colleagues (261) used a full-length version (rN) and truncated versions of PUUV N (rN_1-79_, rN_1-118_, rN_229-327_, rN_1-267_) and aimed to investigate their immunogenic properties upon vaccination and challenge infection of bank voles. Voles were immunized with 50 µg of each vaccine three times three weeks apart and were infected with PUUV two weeks after the last immunization. Immunization with full-length rN, truncated rN_1-79_, rN_1-118_, rN_229-327_, rN_1-267_, or synthetic peptide N_241-270_ induced high levels of anti-PUUV N-specific IgG antibodies, however, these antibodies did not show neutralizing activity. Furthermore, immunization with full-length rN, and truncated rN_1-79_, rN_1-118_, rN_1-267_ fully protected voles against PUUV infection, whereas immunization with rN_229-327_ only partially protected voles against PUUV infection.

Kehm and colleagues (263) used transgenic tobacco and potato plants to express PUUV N protein and immunized New Zealand white rabbits with leaf tissue extracts intramuscularly (IM) and IP four times two weeks apart. When using the collected rabbit antisera in a Western blot, the authors were able to confirm immunogenicity against authentic PUUV N protein.

De Carvalho Nicacio and colleagues (264) immunized bank voles with recombinant nucleoprotein (rN) derived from PUUV, ANDV, DOBV or Topograf orthohantavirus (TOPV) and screened for cross-reactivity and protective efficacy upon challenge infection with PUUV. Bank voles were immunized three times three weeks apart with 50 µg of rN and challenge infected with wild-type PUUV (strain *Kazan*) two weeks after the last immunization. Bank voles do not succumb to PUUV infection and thus, protective efficacy was determined by analyzing lung tissue samples for the presence of viral RNA. Voles immunized with TOPV rN or PUUV rN showed complete protection, whereas with ANDV rN or DOBV rN partial protection was observed. The highest cross-reactivity against PUUV antigen was observed in sera of ANDV rN immunized bank voles, followed by those vaccinated with TOPV rN and DOBV rN.

Dargeviciute and colleagues (265) used the yeast expression system (*Saccharomyces cerevisiae strain FH4C*) to generate recombinant PUUV N, either in its authentic form or fused with a his-tag, and evaluated its immunogenicity in a bank vole challenge model. Voles were immunized with 50 µg of each vaccine three weeks apart and challenge infected with PUUV two weeks after the last immunization. Voles immunized with the authentic PUUV N antigen were only partially protected, whereas immunization with the his-tagged PUUV N antigen fully protected voles. In a subset of experiments, the his-tagged PUUV N antigen was emulsified in alum and bank voles were immunized and infected as described above. Six out of eight voles were completely protected, whereas the remaining two voles showed only partial protection.

In a follow up study, Khattak and colleagues (266) used the PUUV N protein, which was expressed in transgenic tobacco and potato plants, to evaluate the immunogenicity upon oral administration. BALB/c mice were fed with cheese balls containing the air-dried tobacco leaves or with pieces of the potato plants on days 1, 2, 17 and 31. In addition, mice received a booster administration (IP) ten weeks after the first immunization to test memory immune responses. Overall, no anti-PUUV specific antibody responses were induced upon oral administration of the recombinant proteins. Furthermore, the booster immunization did not induce memory immune responses.

Zhao and colleagues designed (268) a multi-epitope-based vaccine based on potential immunodominant B and T cell epitopes from HTNV, PUUV and SEOV Gn/Gc and immunized BALB/c mice once intramuscularly with 100 µg of the multi-epitope vaccine. Cytokine profile analysis of collected splenocytes revealed an increase in IFN-γ, IL-10, and IL-4 until days 31 (IFN-γ, IL-10) and 60 (IL-4), however, the levels of cytokines decreased swiftly until day 90. In addition, low level of neutralizing antibodies against HTNV and SEOV could be detected (neutralization against PUUV was not tested). Furthermore, they observed IgG binding antibody responses against the designed multi-epitope sequence, which increased until day 31 and remained stable until day 90.

### 8.3 DNA based vaccines

Koletzki and colleagues (269) immunized BALB/c mice with plasmid pcDNA3 encoding for the full-length N sequence via the IM route. Serum samples were collected six and eleven weeks after immunization and high titers of PUUV-N specific antibodies could be detected. Further analysis revealed that reactive B cell epitopes are distributed along the whole N protein.

Bucht and colleagues (270) developed modified DNA-based vaccines targeting PUUV N using an intracellular version, a secreted version (addition of an N-terminal secretion signal) and two membrane associated versions (carboxyl (C) -terminal addition of a glycosylphosphatidylinositol (GPI) anchor or a transmembrane (TM) signal) of the protein. BALB/c mice were immunized intramuscularly four times three weeks apart with 50 µg of each vaccine and seroconversion was confirmed in all mice which received the secreted form of PUUV N. In contrary, no seroconversion was observed in mice which received the intracellular form of PUUV N. Immunization with any of the two transmembrane associated forms resulted in partial seroconversion. Furthermore, bank voles were immunized intramuscularly four times three weeks apart with 50 µg of each vaccine and were challenge infected with PUUV (strain *Kazan-wt*). No seroconversion and no protection against PUUV were observed in any mice which were immunized with the intracellular form of PUUV N. Seroconversion and partial protection against PUUV were observed in voles immunized with the secreted, TM and GPI anchor form.

Lindkvist and colleagues (271) generated DNA-based vaccines targeting PUUV N, SEOV N or SNV N and designed full-length or truncated/deleted versions of the respective proteins. BALB/c (cJBom) mice were immunized five times two weeks apart with the DNA vaccines via the gene-gun method and they mainly focused on the analysis of cross-reactive antibody responses as outcome. As expected, they observed high antibody titers towards the corresponding full-length N protein. Interestingly, they observed high cross-reactivity of SNV N serum against PUUV N antigen and a moderate cross-reactivity against SEOV N antigen. When they screened all other possible combinations, only low level of cross-reactivity was observed. In addition, they immunized BALB/c (cJBom) mice with truncated/deleted versions of PUUV N, SEOV N, and SNV N and aimed to determine the location of B cell epitopes, which they identified to be located at the N-terminal part of the N protein.

Spik and colleagues (272) tested the immunogenicity of DNA-based vaccines encoding for the PUUV or HTNV Gn/Gc glycoprotein and evaluated their protective efficacy against HTNV challenge infection in Syrian golden hamsters. The vaccines (single or combined as mixture) were either administrated using the IM (via electroporation) or the intradermal (ID) route (via particle-mediated epidermal delivery (PMED)). Hamsters were immunized three times with 100 µg DNA each three weeks apart using the electroporation approach, with the combined PUUV/HTNV DNA vaccine being administrated either at the same injection site or at separate sites. Hamsters were immunized three times with 5-10 µg of DNA each three to four weeks apart using the PMED approach, with the combined PUUV/HTNV DNA vaccine being administrated 1) separate at adjacent injection sites, 2) by coating both plasmids onto the same gold beads or 3) by coating both plasmids onto different gold beads before mixing together. In addition, a subset of hamsters was infected with HTNV (273) via the IM route and as the animal do not develop clinical symptoms, the level of N-specific antibodies (which is not part of the vaccine) were determined as a surrogate of protection (272). When analyzing the sera four weeks after the last immunization, neutralizing antibodies against PUUV or HTNV were detected for both methods when individual vaccines were administrated. However, when analyzing the sera of hamsters that received both vaccines as a mixture, the elicited level of neutralizing antibodies against both HTNV or PUUV was strongly reliant on the administration mode. A substantial titer of neutralizing antibodies could be detected when immunizing the animals at different injection sites or coating the plasmids on different beads. The same effect could be observed when analyzing the sera of HTNV challenge infected hamsters. The PUUV DNA vaccine alone induced only poor humoral responses (13% for PMED and 38% for electroporation), which was inferior compared to the HTNV DNA vaccine alone (63% for PMED and 88% for electroporation). However, when used as a mixture and administrated at a separate injection site or coated on different gold beads, all hamsters showed protection.

Based on these promising observations, three open-label, single-center phase I studies were conducted, immunizing volunteers with either the PUUV or HTNV DNA vaccine or combined as a mixture using PMED (246), intramuscular electroporation (IM-EP) (277) or intramuscular delivery via the PharmaJet Stratis^®^ needle-free injection system (279). In the first study (246), a total of 27 individuals were immunized three times via the PMED route with 8 µg of the PUUV DNA vaccine, the HTNV DNA vaccine or a mixture of both (half-dose of each), following a 0-1-2 immunization schedule. All vaccines were found to be safe and were well tolerated with no observed severe adverse events related to the study procedures or the vaccines. Serum samples were analyzed for neutralizing antibodies by PRNT_50_ and determined as seropositive if measurable titers were found within at least one of the collected serum samples. Overall, the rate of seroconversion was low, with 30% for the HTNV DNA vaccine group, 44% for PUUV DNA vaccine group, and 56% for the PUUV/HTNV DNA group. In the second study (277), a total of 27 individuals were immunized three times via the IM-EP route with 2 mg of the PUUV DNA vaccine, the HTNV DNA vaccine or combined as a mixture (half-dose each), following a 0-1-2 immunization schedule. Serum samples were analyzed for neutralizing antibodies by PRNT_50_. Seroconversion was observed in 5/9 and 7/9 vaccinees who received all vaccinations with the HTNV or PUUV vaccines, respectively, and in 7/9 vaccinees against PUUV who received the combined vaccine preparation. Interestingly, more individuals responded to the PUUV vaccine than to the HTNV vaccine in the combined vaccine group, however, the three individuals with the highest PRNT_50_ titer against PUUV had also high amounts of neutralizing antibodies directed against HTNV. In the third study (279), a total of 27 individuals were immunized four times via the PharmaJet Stratis^®^ needle-free injection system with 2 mg of the PUUV DNA vaccine, HTNV DNA vaccine or combined as a mixture (half-dose each), following a 0-1-2-6 immunization schedule. Serum samples from 22 individuals were analyzed for neutralizing antibodies using PRNT_50_ and pseudovirion neutralization assay (PsVNA), where titers determined with PRNT_50_ were found to be lower compared to PsVNA titers. Seroconversion was observed in 7/7 and 6/6 individuals receiving the HTNV or PUUV DNA vaccines, respectively, and only in 4/9 individuals who received the combined PUUV/HTNV vaccine. As already observed in the previous study, individuals who received the combined PUUV/HTNV vaccine responded more to PUUV than to HTNV. Cross-reactivity against HTNV was observed in individuals who received the PUUV DNA vaccine and vice versa. In addition, little cross-reactivity against DOBV was observed in samples from all three vaccine groups.

As a follow up, a randomized, double-blinded, phase IIa study was conducted (278), focusing on an optimized, combined PUUV/HTNV DNA vaccine to determine an optimal dose and immunization schedule. A total of 120 subjects were divided into 4 cohorts and subsequently immunized three or four times (0-1-2-6 immunization schedule) with either 2 mg (1 mg per vaccine) or 1 mg (0.5 mg per vaccine) of the PUUV/HTNV DNA vaccine. Cohorts 1 and 3 received four administrations, whereas cohorts 2 and 4 received a phosphate buffered saline vehicle at day 28. Serum samples were screened for neutralizing antibodies by HTNV and PUUV PsVNAs or PRNT_50_. The PUUV/HTNV DNA vaccine induced strong anti-HTNV neutralizing antibodies in the presence of the PUUV vaccine. Overall, only 9% of the subjects did not respond to the PUUV/HTNV vaccine at any point during the study. The second vaccination at day 28 induced higher seropositivity rates in cohorts 1 and 3 compared to the cohorts which received only phosphate buffered saline, however, analysis of subsequent time points could not demonstrate significant differences between three and four vaccinations. Overall, subjects from cohort 3 (four immunizations with 0.5 mg vaccine each) showed the highest rate of seropositivity and highest median neutralizing titers against both PUUV and HTNV.

### 8.4 Virus-like particle vaccines

Ulrich and colleagues (274) generated three chimeric VLP vaccine candidates based on the core antigen of hepatitis B virus which presented PUUV N fragments (N_1-45_, N_38-72_, or N_75-119_) on their surface. Bank voles were immunized subcutaneously three times three weeks apart with 50 µg of each vaccine and challenge infected with PUUV (strain *Kazan*) two weeks after the last immunization. All vaccinated animals showed high titers of N-specific antibodies. Bank voles immunized with VLP carrying fragment N_1-45_ showed the highest protection, followed by N_75-119_. Immunization with VLP carrying fragment N_38-72_ did not induce protective immunity.

### 8.5 Recombinant lentivirus vaccines

Shkair and colleagues (275) developed PUUV candidate vaccines based on microvesicles (MVs) that carry PUUV N and/or PUUV Gn/Gc glycoproteins and evaluated their immunogenicity in C57BL/6 mice. Mice were immunized SC with MVs (15 µg/50 µL) containing PUUV N or PUUV Gn/Gc or both, PUUV N and PUUV Gn/Gc. They detected elevated levels of anti-orthohantavirus-specific IgG in sera of all vaccinated mice at days 14 and 28 post immunization. The highest level of seroconversion was found in the sera of mice vaccinated with MVs carrying both PUUV N and PUUV Gn/Gc. In addition, they confirmed cellular immune responses upon vaccination with all three vaccines by measuring IFN-γ secretion of activated cytotoxic T lymphocytes. Furthermore, they confirmed the induction of cytokines (TNF-α, IL-6, GM-CSF, and G-CSF), which are important to stimulate proliferation and/or differentiation of leukocytes and stimulating phagocytosis by other immune cells, such as macrophages.

In a follow up study, Shkair and colleagues (276) used the developed and characterized PUUV N and PUUV N/G vaccines to screen for specific immunogenic regions of the N protein by using synthesized N peptide fragments. C57BL/6 mice were immunized SC with the two vaccines and collected sera were screened for immunoreactivity towards PUUV N, HTNV N, DOBV N and ANDV N peptides. Vaccination with PUUV N/G induced the selection of more immunogenic PUUV N-specific epitopes compared to PUUV N alone (eleven vs. seven, respectively). In addition, sera of PUUV N vaccinated mice reacted with seven HTNV, two DOBV and two ANDV N peptide fragments, whereas PUUV N/G reacted with only four HTNV N peptide fragments, which were none of the seven HTNV N peptide fragments that reacted with the PUUV N vaccine alone. Overall, the identified reacting and cross-reacting N peptides were located at the N- and C-terminal part of the full-length hantavirus N protein, which are important for the replication process.

## 9 Reverse genetics

Reverse genetics (RG) systems are well-established and highly efficient molecular tools. RG systems allow for replication and transcription of either full-length viral RNA genomes or truncated analogues from a complementary DNA (cDNA), with the aim to produce a full-length infectious viral clone or minigenome systems. Contrary to classical genetics approaches, in which a certain phenotype is analyzed for its causative genotype, RG systems aim to manipulate a viral genotype and analyze the resulting changes in the phenotype (285). One major advantage of using minigenome systems is the possibility to handle and manipulate them under biosafety level (BSL) 1/2 conditions. Especially for working with highly pathogenic viruses, such as *Orthohantaviridae* and *Filoviridae* (e.g., Ebola-virus, Marburg-virus), which require BSL3 and 4 laboratories, respectively, RG offers a convenient way to analyze their pathology, life cycle or molecular biology potentially under lower biosafety requirements if attenuated versions can be generated [for review (286–288)]. In addition, animal studies involving hantaviruses and other highly pathogenic viruses require BSL3 and 4 animal facilities, which are expensive in maintenance and are associated with many restrictions and conditions. Suitable RG systems would potentially allow the efficacy testing of vaccines or therapeutics under lower biosafety requirements.

To obtain recombinant viruses using RG, the first step is the generation of cDNA plasmid intermediates that contain the viral genome or reporter proteins (e.g., to study the function of non-coding regions). The transcription of biologically active molecules is placed under the control of a DNA-dependent RNA polymerase (DdRP), such as RNA polymerase I/II (289) or T7 RNA polymerase (290). Upon transfection of cells with the cDNA plasmids and the co-expression of the respective DdRP, an unencapsidated genomic RNA is transcribed. However, a hallmark of negative-strand RNA viruses is the necessity of viral RNA to be encapsidated by the nucleoprotein to serve as a template for the viral polymerase (286). Therefore, the nucleoprotein has to be co-expressed and once encapsidated, other ribonucleoprotein components, that are provided by either helper virus co-infection or helper plasmid co-transfection, recognize the genomic RNA and initiate replication and transcription into mRNAs. Subsequently, all viral proteins required to start the viral replication cycle are translated, which ultimately leads to the generation of infectious viruses (286).

Reverse genetics systems have been established for several *Bunyavirales* (*Peribunyaviridae*, *Nairoviridae* or *Arenaviridiae*), based on either the bacteriophage T7 RNA polymerase system or RNA polymerase I/II system (291). However, attempts to establish RG systems for hantaviruses were only partially successful, but mostly failed (292). In the late 1990s, Welzel and colleagues (293) efficiently expressed PUUV and HTNV N in mammalian cells, a first step towards the establishment of an eukaryotic system for hantavirus reverse genetics. Flick and colleagues (294) successfully established the first HTNV minigenome, thereby demonstrating the expression of a functional recombinant hantavirus polymerase and the rescue of HTNV minigenomes without superinfection with infectious hantavirus, allowing for handling of the recombinant viral clones outside a BSL3 facility. However, the established system was not further used, suggesting suboptimal tractability (295). The first minigenome system for ANDV was described more than 10 years ago by Brown and colleagues (292), however, unsuccessful expression of the L-protein and lack of reproducibility were observed. Infectious virus has not been rescued so far.

Overall, successful and tractable RG systems for hantaviruses are urgently required for a better understanding of certain molecular pathways and to establish effective antivirals and vaccines. However, to develop effective RG systems for hantaviruses, several obstacles and hurdles have to be overcome as shown by the previous failed attempts (292, 294): (I) the type of promoter (weak or strong) used to express the viral genome, (II) the presence of a potential cryptic promoter in the 3´ non-coding regions, (III) hantavirus- and/or host-cell specific factors that might inhibit rescue of an infectious clone, (IV) the determination/consideration of a correct biological ratio for the expressed viral proteins, (V) the choice of suitable cell lines for transfection; e.g., Vero E6 cells are suitable for viral infection and propagation, but BHK-21 cells show a higher transfection efficiency, (VI) the potential need of cellular factors that only exist in the reservoir but are not present in available cell lines.

Intra-and inter-lineage reassortment events of naturally circulating strains are reported frequently [for review: (296)] and *in vitro* reassortment systems are a suitable alternative to reverse genetic systems for culturing hantavirus species and analyze e.g., growth characteristics or innate immune responses. Cell cultures are co-infected with two closely related hantavirus species which allows the two viruses to exchange genomic segments. Subsequently, the infected cell cultures are screened to identify novel reassortant viruses. Interestingly, current attempts were only successful in exchanging the M segment between two hantavirus species, indicating that certain species-specific determinants (297) inhibit heterologous reassortment. Furthermore, the high instability of reassortant viruses suggests a co-requirement of L and S segments from the same hantavirus species (298). The RdRp which is encoded by the L segment interacts with the encapsidated RNA in a sequence specific manner, and interaction with the nucleocapsid protein (encoded by the S segment) is required for viral replication and transcription (299). In the late 1990s, Rodriguez and colleagues (83) generated reassortant viruses by co-infecting SNV and Black Creek Canal virus (BCCV), thereby obtaining virus plaques that appeared diploid, containing S or M segments originating from parental SNV and BCCV. However, most of these diploid virus genotypes were unstable and only one reassortant virus, based on L and S segments from BCCV, and M segment from SNV appeared stable. McElroy and colleagues (298) recovered a reassortant virus (SAS-11) based on SNV L and S segments and ANDV M segment. Other combinations, e.g., ANDV L and S segments and SNV M segment turned out to be unstable and were lost during plaque isolation. SAS-11 showed similar plaque morphology and growth characteristics as ANDV, but failed to induce a lethal infection in Syrian hamsters. Handke and colleagues (297) generated a reassortant virus (PHPUV) by co-infecting PUUV and Prospect Hill virus (PHV), which contained the PUUV M segment and the PHV L and S segments. PHPUV showed growth characteristics and ability to stimulate innate immune responses *in vitro* similar to the parental PHV.

## 10 Discussion and future perspectives

Nephropathia epidemica is an important and significant disease in Europe that is caused by PUUV infections. While thousands of cases are reported every year, it is likely also underdiagnosed and underreported. In recent years, major advances to a better understanding of this viral infection have been made. These include the elucidation of the structure of the viral surface glycoproteins and the discovery of broadly protective hantavirus mAbs. However, much remains to be done. A reverse genetics system for hantaviruses in general has not been established and is urgently needed to better understand PUUV biology. A reverse genetics system could also be used to rationally design live attenuated vaccines. In addition, better animal models for PUUV that more closely reflect human disease and can be used for evaluation of therapeutics and prophylactics are also urgently needed. Furthermore, no human vaccines or therapeutics for PUUV are available. MAbs as therapeutic candidates have been developed but still need to enter clinical development. In addition, no PUUV vaccines are available for human use. While several vaccine candidates already exist, mRNA vaccine development also opens up exciting new avenues for the development of PUUV vaccines. However, while thousands of cases occur every year, it will potentially be difficult to perform late-stage clinical trials to establish efficacy of vaccines or treatments. We do believe that this is possible by focusing on risk groups and areas in Europe where clinicians are very experienced with diagnosing the disease, like parts of Sweden, Finland, Germany, Austria or Slovenia. While there is certainly a medical need, it is also unclear if there is a business case for commercial entities to develop vaccines or therapeutics for PUUV infections. It is likely that these interventions have to be developed through public-private partnerships and with the help of public funding bodies like the European Union.
