# Supplementary material for: Puumala orthohantavirus: prevalence, biology, disease, animal models and recent advances in therapeutics development and structural biology
Source: Front Immunol. 2025 May 8;16:1575112. doi: 10.3389/fimmu.2025.1575112 (PMC12095308; doi:10.3389/fimmu.2025.1575112)
Supplement: Supplementary file 1 [file DataSheet1.docx]

Supplementary Material

# Supplementary Data

Tscherne A *et al.*

Puumala Orthohantavirus: Prevalence, biology, disease, animal models and recent advances in therapeutics development and structural biology

**Additional clinical features of PUUV infection**

Beside clinical features of HFRS many PUUV infected patients have abdominal pain, nausea, vomiting, and diarrhea (145, 299). Hemorrhagic gastropathy was common in one case series (300), and hematemesis as well as melena may sometimes occur. Spotty hemorrhages have been seen in one patient undergoing colonoscopy suggesting that the entire gastrointestinal tract is involved in PUUV infections. Histology of lesions in the gastrointestinal tract showed tissue edema, explained by the underlying pathophysiological mechanism of capillary leakage (300). PUUV infections with gastrointestinal signs and symptoms including nausea, vomiting, abdominal tenderness and abdominal pain might pretend acute abdomen. Cases of PUUV infections undergoing exploratory laparotomy due to clinically suspected acute abdomen have been described (301-303). In radiological examination of the abdomen, retroperitoneal fat stranding, perirenal fascial thickening and/or perirenal fat stranding have been described in most patients with acute PUUV infection (304). Acute myopericarditis has been rarely diagnosed (<3%) in a previous cohort of NE patients (146). However, in another study more than half of hospital-treated NE patients had transient abnormal cardiac findings examined by electrocardiograms and echocardiography (305). PUUV infection might also result in disseminated intravascular coagulopathy, indicated by thrombocytopenia and hemorrhages. In laboratory examinations, elevation of prothrombin fragments and D-dimer indicate increased thrombin formation as well as fibrinolytic activity (306, 307).

Headache, insomnia, vertigo, nausea, anxiety and blurred vision are common in PUUV infections and indicate involvement of the central nervous system and the eyes (308). Altered mental status and seizures have also been described. Most of the ocular and CNS related symptoms are reversible, but serious complications such as pituitary hemorrhage and encephalitis have been reported (302, 308). Involvement of the eyes is common in PUUV infection and often leads to reduced vision, transient myopia or myopic shift (50 to 78%) and changes in ocular dimensions (309, 310). Other ocular findings include acute glaucoma attacks, decrease in intraocular pressure, anterior uveitis, lid edema, conjunctival chemosis, conjunctival hemorrhages, retinal edema and hemorrhages, diplopia, and anisocoria (308). Myopic shift has been considered a pathognomonic sign of NE and HFRS but rates of ocular involvement varied. In one study, blurred vision and myopic shift were independent of NE severity (310). Alterations of various hormones might occur during acute PUUV infection and persistent hormone deficits after recovery from the acute phase of the PUUV infection have been described (311). Previously, individual cases of hypopituitarism in patients with orthohantavirus infections (mainly PUUV) have been summarized, but the duration of pituitary gland dysfunction has been reported only for one PUUV case and lasted 5 months (312, 313). In another case series, autopsy showed hemorrhagic pituitary gland positive for PUUV antigen in one patient and panhypopituitarism with hypophyseal hemorrhage diagnosed with magnetic resonance in the other two patients (314).

The severity of acute PUUV infection does not predict the long-term outcome of the patients (315). Even severe courses of PUUV infections resulting in acute kidney injury have a favorable outcome (316). It has been reported that patients recovering from NE still had slightly more proteinuria, higher glomerular filtration rate (GFR), and higher systolic blood pressure compared with healthy controls (317). However, after 10 years of follow up, glomerular hyperfiltration and previously detected elevated proteinuria had disappeared (318). It has been hypothesized that NE may predispose some patients to the development of hypertension.

**Laboratory results in patients with PUUV infection**

In laboratory examinations, thrombocytopenia (the most common laboratory finding in HFRS and NE), leukocytosis, proteinuria, hematuria, elevated serum C-reactive protein, and creatinine levels are typical findings in patients with PUUV infection. In patients with oliguria or anuria, elevated serum creatinine levels, proteinuria, and hematuria, which are then followed by polyuria in the second week after the onset of infection can be detected (319). Previously, glucosuria has been described as a potential biomarker for severe courses (320). Furthermore, low absolute lymphocyte counts and dyspnea were associated with a severe course of PUUV infection (162).

**References**

145. Mustonen J, Mäkelä S, Outinen T, Laine O, Jylhävä J, Arstila PT, et al. The Pathogenesis of Nephropathia Epidemica: New Knowledge and Unanswered Questions. *Antiviral research* (2013) 100(3):589–604. Epub 12.10.2013. doi: 10.1016/j.antiviral.2013.10.001.

146. Mustonen J, Brummer-Korvenkontio M, Hedman K, Pasternack A, Pietilä K, Vaheri A. Nephropathia Epidemica in Finland: A Retrospective Study of 126 Cases. *Scandinavian journal of infectious diseases* (1994) 26(1):7–13. doi: 10.3109/00365549409008583.

162. Hatzl S, Posch F, Linhofer M, Aberle S, Zollner-Schwetz I, Krammer F, Krause R. Poor Prognosis for Puumala Virus Infections Predicted by Lymphopenia and Dyspnea. *Emerging Infectious Diseases* (2023) 29(5):1038–41. doi: 10.3201/eid2905.221625.

299. Lähdevirta J. Nephropathia Epidemica in Finland. A Clinical Histological and Epidemiological Study. *Annals of clinical research* (1971) 3:1–54.

300. Nuutinen H, Vuoristo M, Färkkilä M, Kahri A, Seppälä K, Valtonen V, et al. Hemorrhagic Gastropathy in Epidemic Nephropathy. *Gastrointestinal endoscopy* (1992) 38(4):476–80. doi: 10.1016/s0016-5107(92)70480-5.

301. Bauer PK, Krause R, Fabian E, Aumüller M-L, Schiller D, Adelsmayr G, et al. Clinical-Pathological Conference Series from the Medical University of Graz: Case No 172: A 45-Year-Old Truck Driver with Fever, Vomiting, Thrombocytopenia and Renal Failure. *Wiener Klinische Wochenschrift* (2021) 133(21-22):1222–30. doi: 10.1007/s00508-021-01921-z.

302. Krause R, Aberle S, Haberl R, Daxböck F, Wenisch C. Puumala Virus Infection with Acute Disseminated Encephalomyelitis and Multiorgan Failure. *Emerging Infectious Diseases* (2003) 9(5):603–5. doi: 10.3201/eid0905.020405.

303. Bennedbaek FN, Søe KL. Nephropathia Epidemica. Hantavirusnephritis--En Differentialdiagnose Til Akut Abdomen. *Ugeskrift for laeger* (1994) 156(43):6392–3305.

304. Lebecque O, Falticeanu A, Mulquin N, Dupont M. Abdominal Ct Findings in Puumala Hantavirus-Infected Patients. *Abdominal radiology (New York)* (2022) 47(7):2552–9. Epub 20.04.2022. doi: 10.1007/s00261-022-03467-8.

305. Makela S, Kokkonen L, Ala-Houhala I, Groundstroem K, Harmoinen A, Huhtala H, et al. More Than Half of the Patients with Acute Puumala Hantavirus Infection Have Abnormal Cardiac Findings. *Scandinavian journal of infectious diseases* (2009) 41(1):57–62. doi: 10.1080/00365540802502629.

306. Laine O, Mäkelä S, Mustonen J, Huhtala H, Szanto T, Vaheri A, et al. Enhanced Thrombin Formation and Fibrinolysis During Acute Puumala Hantavirus Infection. *Thrombosis research* (2010) 126(2):154–8. Epub 01.07.2010. doi: 10.1016/j.thromres.2010.05.025.

307. Koskela S, Mäkelä S, Strandin T, Vaheri A, Outinen T, Joutsi-Korhonen L, et al. Coagulopathy in Acute Puumala Hantavirus Infection. *Viruses* (2021) 13(8). doi: 10.3390/v13081553.

308. Hautala N, Partanen T, Kubin A-M, Kauma H, Hautala T. Central Nervous System and Ocular Manifestations in Puumala Hantavirus Infection. *Viruses* (2021) 13(6). doi: 10.3390/v13061040.

309. Hautala N, Kauma H, Vapalahti O, Mähönen S-M, Vainio O, Vaheri A, Hautala T. Prospective Study on Ocular Findings in Acute Puumala Hantavirus Infection in Hospitalised Patients. *The British journal of ophthalmology* (2011) 95(4):559–62. Epub 01.08.2010. doi: 10.1136/bjo.2010.185413.

310. Theiler G, Langer-Wegscheider B, Zollner-Schwetz I, Valentin T, Hönigl M, Schnedl W, Krause R. Blurred Vision and Myopic Shift in Puumala Virus Infections Are Independent of Disease Severity. *Clinical microbiology and infection : the official publication of the European Society of Clinical Microbiology and Infectious Diseases* (2012) 18(10):E435-7. Epub 22.08.2012. doi: 10.1111/j.1469-0691.2012.03997.x.

311. Mäkelä S, Jaatinen P, Miettinen M, Salmi J, Ala-Houhala I, Huhtala H, et al. Hormonal Deficiencies During and after Puumala Hantavirus Infection. *European journal of clinical microbiology & infectious diseases : official publication of the European Society of Clinical Microbiology* (2010) 29(6):705–13. Epub 16.04.2010. doi: 10.1007/s10096-010-0918-y.

312. Jost C, Krause R, Graninger W, Weber K. Transient Hypopituitarism in a Patient with Nephropathia Epidemica. *BMJ Case Reports* (2009) 2009. doi: 10.1136/bcr.02.2009.1538.

313. Bhoelan S, Langerak T, Noack D, van Schinkel L, van Nood E, van Gorp ECM, et al. Hypopituitarism after Orthohantavirus Infection: What Is Currently Known? *Viruses* (2019) 11(4). doi: 10.3390/v11040340.

314. Hautala T, Sironen T, Vapalahti O, Pääkkö E, Särkioja T, Salmela PI, et al. Hypophyseal Hemorrhage and Panhypopituitarism During Puumala Virus Infection: Magnetic Resonance Imaging and Detection of Viral Antigen in the Hypophysis. *Clinical infectious diseases : an official publication of the Infectious Diseases Society of America* (2002) 35(1):96–101. Epub 06.06.2002. doi: 10.1086/340859.

315. Miettinen MH, Mäkelä SM, Ala-Houhala IO, Huhtala HSA, Hurme MA, Kööbi T, et al. The Severity of Acute Puumala Hantavirus Infection Does Not Predict the Long-Term Outcome of Patients. *Nephron Clinical practice* (2010) 116(2):c89-94. Epub 21.05.2010. doi: 10.1159/000314657.

316. Outinen TK, Mäkelä S, Clement J, Paakkala A, Pörsti I, Mustonen J. Community Acquired Severe Acute Kidney Injury Caused by Hantavirus-Induced Hemorrhagic Fever with Renal Syndrome Has a Favorable Outcome. *Nephron* (2015) 130(3):182–90. Epub 26.06.2015. doi: 10.1159/000433563.

317. Mäkelä S, Ala-Houhala I, Mustonen J, Koivisto AM, Kouri T, Turjanmaa V, et al. Renal Function and Blood Pressure Five Years after Puumala Virus-Induced Nephropathy. *Kidney international* (2000) 58(4):1711–8. doi: 10.1046/j.1523-1755.2000.00332.x.

318. Miettinen MH, Mäkelä SM, Ala-Houhala IO, Huhtala HSA, Kööbi T, Vaheri AI, et al. Ten-Year Prognosis of Puumala Hantavirus-Induced Acute Interstitial Nephritis. *Kidney international* (2006) 69(11):2043–8. doi: 10.1038/sj.ki.5000334.

319. Iheozor-Ejiofor R, Vapalahti K, Sironen T, Levanov L, Hepojoki J, Lundkvist Å, et al. Neutralizing Antibody Titers in Hospitalized Patients with Acute Puumala Orthohantavirus Infection Do Not Associate with Disease Severity. *Viruses* (2022) 14(5). doi: 10.3390/v14050901.

320. Tietäväinen J, Mantula P, Outinen T, Huhtala H, Pörsti IH, Niemelä O, et al. Glucosuria Predicts the Severity of Puumala Hantavirus Infection. *Kidney International Reports* (2019) 4(9):1296–303. doi: 10.1016/j.ekir.2019.05.770.
